# Categorical Exploratory Data Analysis: From Multiclass Classification and Response Manifold Analytics Perspectives of Baseball Pitching Dynamics

**DOI:** 10.3390/e23070792

**Published:** 2021-06-22

**Authors:** Fushing Hsieh, Elizabeth P. Chou

**Affiliations:** 1 Department of Statistics, University of California at Davis, Davis, CA 95616, USA; 2 Department of Statistics, National Chengchi University, Taipei 116, Taiwan; eptchou@g.nccu.edu.tw

**Keywords:** multiclass classification, categorical exploratory data analysis, PITCHf/x

## Abstract

All features of any data type are universally equipped with categorical nature revealed through histograms. A contingency table framed by two histograms affords directional and mutual associations based on rescaled conditional Shannon entropies for any feature-pair. The heatmap of the mutual association matrix of all features becomes a roadmap showing which features are highly associative with which features. We develop our data analysis paradigm called categorical exploratory data analysis (CEDA) with this heatmap as a foundation. CEDA is demonstrated to provide new resolutions for two topics: multiclass classification (MCC) with one single categorical response variable and response manifold analytics (RMA) with multiple response variables. We compute visible and explainable information contents with multiscale and heterogeneous deterministic and stochastic structures in both topics. MCC involves all feature-group specific mixing geometries of labeled high-dimensional point-clouds. Upon each identified feature-group, we devise an indirect distance measure, a robust label embedding tree (LET), and a series of tree-based binary competitions to discover and present asymmetric mixing geometries. Then, a chain of complementary feature-groups offers a collection of mixing geometric pattern-categories with multiple perspective views. RMA studies a system’s regulating principles via multiple dimensional manifolds jointly constituted by targeted multiple response features and selected major covariate features. This manifold is marked with categorical localities reflecting major effects. Diverse minor effects are checked and identified across all localities for heterogeneity. Both MCC and RMA information contents are computed for data’s information content with predictive inferences as by-products. We illustrate CEDA developments via Iris data and demonstrate its applications on data taken from the PITCHf/x database.

## 1. Introduction

The author of the well-known 1977 book Exploratory Data Analysis (EDA) [1], John W. Tukey in his 1962 paper [2] The future of Data Analysis discussed fundamental principles and made clear-cut arguments for data analysis as a scientific discipline. Among many facts, he emphasized that data analysts must put “reliance upon the test of experience as the ultimate standard of validity”. He further wrote that: “Data analysis is a larger and more varied field than inference, or incisive procedures, or allocation.”

This statement rings much louder now than ever before in this big data era. When a system of interest is somehow complex and being embraced by a big dataset, the goal of data analysis is naturally data’s full information content for system understanding. Many tasks can be performed as by-products based on such information content.

Within the 60 years from 1962, the data visualization technique is the most visible kind of effort developed for carrying out EDA in the literature [3,4,5]. Some attempts sparsely appeared in the literature that intend to incorporate EDA into the Bayesian framework [6]. Thus far, we have seen neither well-developed resolutions for major problems listed in Tukey’s paper, including the multiple response problem, nor unified fundamental concepts and computational paradigms as principles of data analysis. Although data analysis nowadays has been widely permeating and drastically expanding in all sciences, one factual sign is continuously blinking: data analysis is still underdeveloped [5,7].

One direct and clear view of such underdevelopment is seen when analyzing a real database from a somehow known complex physical system. Physics tells us the principles that are underlying the system and how they work. However, we often lack knowledge of how these principles realistically couple and link together in working out details. The phrase “The devil is in the detail” seems to capture what is precisely missing in data analysis. Can data analysts discover such principles and missing details of their linkages? Would our data analysis offer a complete understanding of a system of interest? We attempt positive answers to both questions through real complex systems in this paper.

The major real-world complex system considered here is the Major League Baseball’s (MLB) pitching dynamics. In the USA, MLB has been recording and storing every single pitch in its 30 stadiums into its public available PITCHf/x database since 2006. This is one of the best-maintained databases in the world and one very important database for the evolutions of thousands of pitchers’ aerodynamics and biomechanics of pitching. The importance of this database is far beyond baseball as a sport in this big data era since it adds about 700k pitches, each of which is more than 30D data, every season. In this paper, we focus on three complex systems defined by three sets of selected pitchers’ three pitching types.

From the physics perspective, these complex systems involve Newtonian laws of forces, as well as Magnus force of spin [8]. Both forces govern a baseball trajectory. We want to compute details of pitching dynamics contained in the PITCHf/x database without invoking differential equations from physics and aerodynamics literature. Such computable details would make us see which factors make a pitch move and curve the way it does and understand pitchers’ idiosyncratic characteristics. Here, well-patched details mean multiscale and global-to-local pattern information illuminating how the principles work in concert. We collectively term such details as data’s “information content”.

Here, PITCHf/x’s information content regarding pitching dynamics contains at least two major perspectives: (1) what factors can efficiently characterize a pitcher’s pitches; and (2) how the underlying physical principles work. The first perspective is one major classification topic, called multiclass classification (MCC) in machine learning (ML). The second one is the topic called response manifold analytics (RMA) for solving multiple response problems. Here, a manifold would naturally appear for depicting how a group of possibly highly associative response features or variables globally links to a major group of covariate features. Upon this manifold, other minor groups of covariate features can also involve locally, not globally. This manifold-based topic is not yet well developed in the statistics and ML literature.

Further in PITCHf/x example, the response and covariate features have a clear spatial-temporal separation. Covariate features are measured at the pitcher’s mound, while response features are measured near the home plate. Thus, they are not exchangeable. Although RMA is exactly one physical multiple response problem here, its manifold framework is seemingly universal when continuous features of physical or mechanical mechanisms are intertwined and linked. We expect that RMA’s information content should bear with principles on global and large scales and reveals heterogeneous effects on local and fine scales. Data’s deterministic and stochastic structures are all presented through visible and explainable graphic displays based on such information content. These computed structures, in turn, serve as bases for our inferential decision-making. In this fashion, our data analysis indeed coherently reflects the quoted statement at the beginning of this section.

As the first phase of our CEDA developments, we consider only quantitative covariate features under both settings of MCC and RMA here. As such, the locality concept is either based on hypercubes or *k*-nearest neighbors (KNN). In a separate report, the second phase of CEDA is developed to unify both topics simultaneously and accommodate categorical covariate features. Throughout these two phases of CEDA developments, the concepts and devices of histogram, contingency table, and mutual conditional entropy (MCE) matrix constantly play fundamental and critical roles.

We organize this paper as follows. In Section 2, we use three contingency tables based on Iris data to depict concrete ideas of mixing geometry and previews of information contents under MCC and RMA, respectively. In Section 3, we discuss the information content of the PITCHf/x complex system from the perspectives of MCC and RMA. In Section 4 and Section 5, our CEDA-based computational developments are proposed and demonstrated under the MCC and RMA settings, respectively. Conclusions and remarks on our CEDA computing are given in Section 6.

## 2. Tabular Views of Mixing Geometry and Previews of MCC and RMA Information Contents

Before our CEDA developments under the MCC and RMA settings, a collection of computational concepts and devices used in this paper are illustrated with the well-known and straightforward Iris data example. These concepts and devices in increasing order of complexity include possibly gapped histogram, contingency table, directional and mutual conditional entropy-based associations, mixing geometry in $R^{k}$ with various dimensions *k*(≥2), and information contents of MCC and RMA.

**Figure 1:**
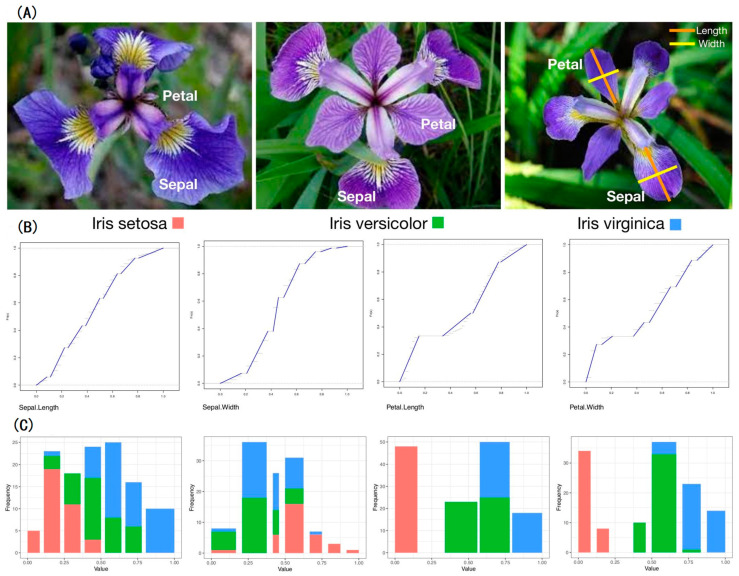
(**A**) Threespecies of Iris: Setosa (red), Versicolor (green), and Virginica (blue); (**B**) four features’ possibly gapped histograms; and (**C**) corresponding piecewise linear approximations on empirical distribution functions.

The Iris dataset from the UCI Machine Learning Data Repository consists of three Iris species: Setosa, Versicolor, and Virginica. Figure 1 (top) presents the three pictures of Iris. Four features of continuous measurements are sepal length and width and petal length and width. This dataset consists of 50 species-labeled 4D vectors. First, each feature is individually categorized via a possibly gapped histogram constructed based on 150 1D data points (see details in [9]). Such histograms reveal unequal bin widths and possible presences of gaps, as shown in Figure 1 (bottom). The underlying characteristic is that each possibly gapped histogram provides a low-complexity piecewise linear approximation to the high-complexity empirical distribution function, as shown in Figure 1 (middle). Such a histogram indeed reveals the authentic categorical nature underlying a continuous feature.

**Figure 2:**
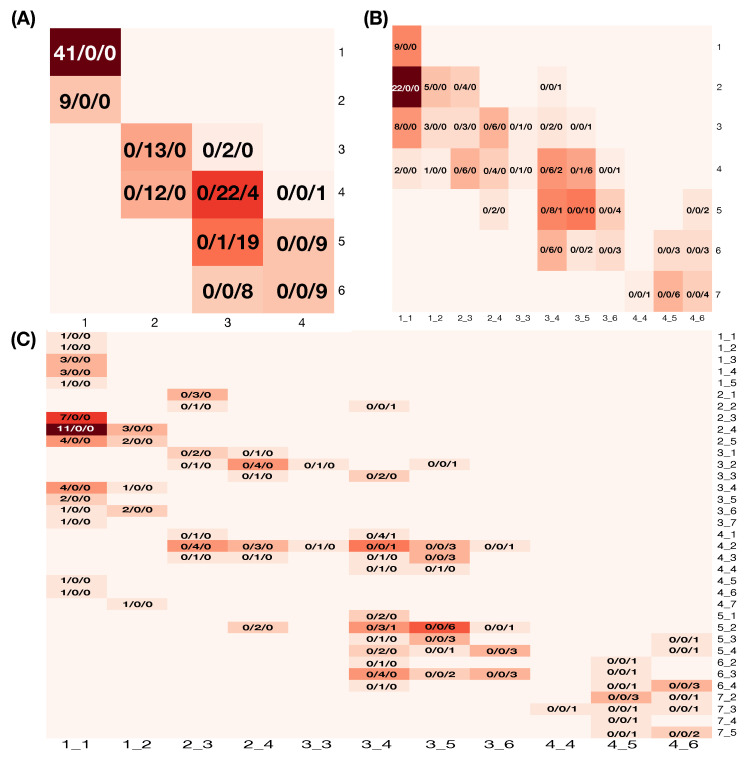
Three contingency tables (row-vs.-column) for mixing geometries: (**A**) petal width-vs.-petal length; (**B**) sepal length-vs.-(petal length, petal width); and (**C**) (sepal length, sepal width)-vs.-(petal length, petal width). Within each cell, the triplet of numbers is: #Setosa/#Versicolor/#Virginica.

A contingency table is framed by two histograms on the row-axis and column-axis, respectively. For instance, a contingency table for petal width-vs.-petal length is given in Figure 2A. Such a contingency table explores and exhibits two features’ potential nonlinear associative relation since each row or column defines a conditional categorical random variable with a probability vector approximated by its corresponding vector of proportions. With such categorical nature, for example, on a given row, the Shannon entropy of cell proportions across all column categories measures a degree of associative uncertainty in the conditioning on the row-specific category of row variables. This row-specific Shannon entropy is then re-scaled to the Shannon entropy of the vector of proportions of column-sums. This ratio reveals specifically the effect of conditioning with respect to this row-specific category. A smaller ratio means a higher conditioning effect.

By carrying out computations of such re-scaled row-specific entropy-ratio across all rows, the directed row-to-column associative measure is calculated as the weighted sums of row-specific entropy-ratios. This directional association is invariant with respect to row-wise and column-wise permutations onto the contingency table. Thus, it can accommodate all nonlinear associations. Further, the lower value of this directional conditional entropy means that the row-variable indeed has higher predictive power for the outcomes of the column-variable (see details and merits in [10]).

**Figure 3:**
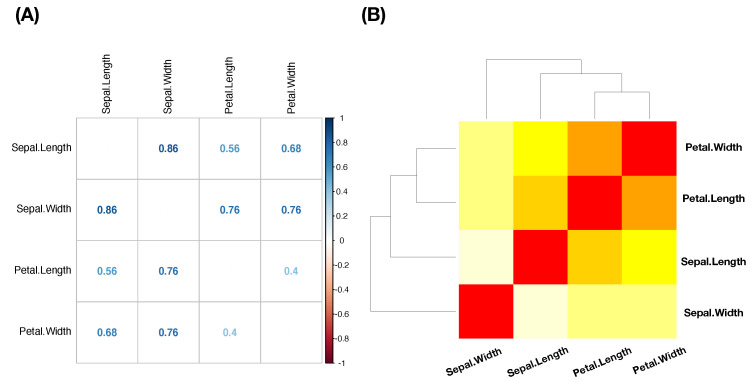
(**A**) Mutualconditional entropy matrices and (**B**) marked synergistic feature-groups on its heatmap.

The column-to-row associative measure is likewise calculated via column-specific Shannon entropies. Then, by simply averaging the two directed entropies, row-to-column and column-to-row, we arrive at the so-called mutual conditional entropy (MCE) of these two features or variables. Then, MCEs are evaluated for all possible pairs and collected into an MCE matrix, as illustrated in Figure 3A. By using such an MCE matrix as a “distance” matrix, we apply the hierarchical clustering (HC) algorithm on the four feature-nodes and result in a hierarchical clustering (HC) tree. Further, by superimposing this HC-tree on the row and column axes of the MCE matrix, we derive a so-called heatmap.

Such a heatmap typically provides multiscale compositions of feature clustering or groupings, as s shown in Figure 3B. These compositional patterns in a heatmap collectively become one platform for obtaining explainable and visible computational results. For instance, the feature-pair (petal length, petal width) is highly associated. This pair then is grouped with sepal length to become a feature triplet. Hence, a MCE heatmap offers significant efficiency in feature selection.

Under the MCC setting, the mixing geometry of the three species of Iris pertaining to the feature-pair (petal width, petal length) is seen in the corresponding contingency table, as shown in Figure 2A. Each cell is coded with the triplet of counts: #Setosa/#Versicolor/#Virginica. Upon the mixing geometry of the three point-clouds in $R^{2}$, we see that only the cells (4,3) and (5,3) are occupied by two species: Versicolor and Virginica. Nine out of 11 occupied cells exclusively belong to one single species. Together, these pieces of information imply that the feature-pair (petal width, petal length) can effectively classify these three species with minor errors. Can we improve upon this feature-pair’s classification performance?

To answer this question, we build a large contingency table with 11 occupied cells in Figure 2A being coded and arranged on the column axis, while the 7 bins of the histogram of sepal length being arranged on the row axis, as shown Figure 2B. On the column 3_5 (pertaining to the aforementioned cell (5,3)), we see clear improvements, while the improvements pertaining to the aforementioned cell (4,3) on the column 3_4 are mild. These improvements indicate that information of sepal length does improve upon the classification capability of (petal width, petal length). Could significant improvements be achieved by incorporating the feature-pair (sepal length, sepal width)?

To explore such a possibility, we build a further expanded 35×11 contingency table with the row-axis having all 35 occupied cells found within the 7×7 contingency table of (sepal length, sepal width), as shown in Figure 2C. All cells on the column 3_5 are all occupied by single species, while there are only two cells on the column 3_4 that are occupied by two species. We recognize that we only need to focus on the two specific columns. This recognition indeed leads to the concept of a chain of complementary feature-groups. We look at focal regions of mixing geometry through multiple perspectives of feature-groups in a serial fashion. Ideally, this chain of complementary feature-groups would constitute the MCC information content.

Under the RMA setting, a natural manifold is based on the four features in this Iris dataset. If we take (petal length, petal width) as response feature-pair and (sepal length, sepal width) as covariate feature-pair, then this manifold should reveal the response-to-covariate associative relations. Although this 4D manifold certainly is not visible in $R^{3}$, some key associative patterns are visible through the 35×11 contingency table, as shown in Figure 2C. This table indeed depicts a categorized 4D manifold with 4D hypercubes as its cells. It is observable that each row has only a few occupied cells. This fact implies that covariate feature-pair (sepal length, sepal width) indeed plays the role of a major factor in predicting a region of the response feature-pair (petal length, petal width). Another important observation is that, if the Iris species is taken as an extra categorical covariate variable, this species variable will play a minor factor since it indicates which columns are more proper for predicting the response pair at each given row. Such major and minor factors are crucial for information content under the RMA setting.

The above tabular views of mixing geometries across three contingency tables and the categorized manifold via Iris data would further motivate our CEDA developments for MCC and RMA, which are fully demonstrated through PITCHf/x data in the rest of this paper.

## 3. PITCHf/x Complex Systems from Perspectives of MCC and RMA

By excluding features related to baseball gaming results, such as “ball-and-strike”, “zone number” and “batting results”, we exclusively select 19 features contained within the PITCHf/x database that are primarily measured and engineered toward pitching dynamics. These 19 features are either biomechanical or physical. The biomechanical features are those measured related to pitching gestures at the moment of pitching on the pitcher’s mound, such as horizontal and vertical coordinates of a releasing point {“x0”, “z0”}; x−, *y*- and z− directional releasing speeds {“vx0”, “vy0”, “vz0”}; spin created by using fingers sliding against skin of baseball {“spin_dir”, “spin_rate”}; and starting speed {“start_speed”}. Physical features refer to those measured before arriving at the home plate, e.g., horizontal and vertical movements {“pfx_x”, “pfx_z”}; degree of curve in trajectory {“break_length”, “break_angle”}; accelerations {“ax”, “az”}; and final speed {“end_speed”}. These biomechanical and physical features are temporally and spatially separated. Such separations imply causal effects within all pitcher-specific pitching dynamic systems. Thus, manifestations of such effects are of primary interest in this paper.

Even though pitching dynamics is primarily governed by Newtonian physics, different pitch-types seemingly reveal their distinct physical and biomechanical characteristics and distinctions. Thus, we divide pitching dynamics here according to three pitch-types: fastball, slider, and curveball. These three pitch-types are significantly different in speed and spin. Such intrinsic distinctions are to be illustrated through graphic displays. Throughout this paper, computational results are represented via graphic displays to reveal many visible aspects of information content regarding each pitch-type’s specific dynamics. Such displays amount to confirm that distinct feature sets manifest different perspectives of data’s information content.

**Figure 4:**
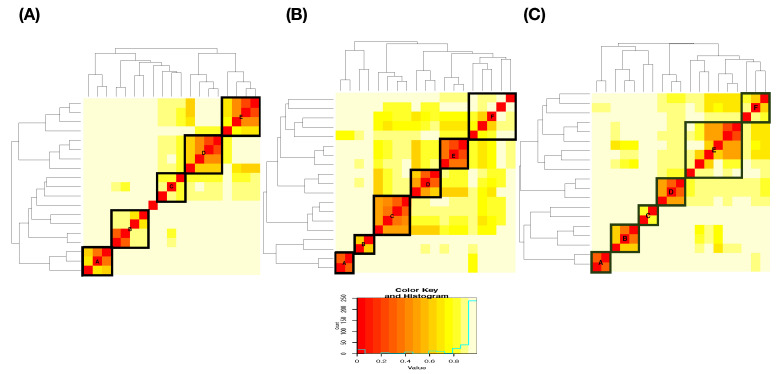
Three mutual conditional entropy matrices with marked synergistic feature-sets: (**A**) slider; (**B**) curveball; and (**C**) fastball.

We begin with building a roadmap for CEDA. As illustrated in the previous section, this roadmap is a heatmap of 19×19 mutual conditional entropy (MCE) matrix. Within this heatmap, we visualize multiscale block patterns upon the 19×19 matrix lattice, as shown in Figure 4A–C for three pitch-types fastball, curveball, and slider, respectively. Such feature-group is identified via an evident block within a heatmap and collectively stands for either a biomechanical or a physical mechanism within the pitching dynamics. Since most of these groups simultaneously embrace temporally and spatially separated features, it is a natural sign of scientific intelligence embraced within a real-world system.

Here, we individually point out and briefly discuss these mechanisms across the three heatmaps. It is noted that memberships of these feature-groups vary only slightly across the three pitch-types. Such a collection of mechanisms intuitively should be essential parts of information contained in the PITCHf/x database. Below, we list four feature-triplets accommodating such mechanisms and then explicitly show them via graphic displays:

**a:**  [Biomechanics] “z0”, “x0” and “vx0”;
**b:**  [Biomechanics and Laws of forces] “x0” and “start_speed” toward “end_speed”;
**c:**  [Biomechanics and Laws of forces] “spin_rate” and “start_speed” toward “end_speed”; and
**d:**  [Magnus effect] “spin_dir” toward {“pfx_x”, “pfx_z”}.

**Figure 5:**
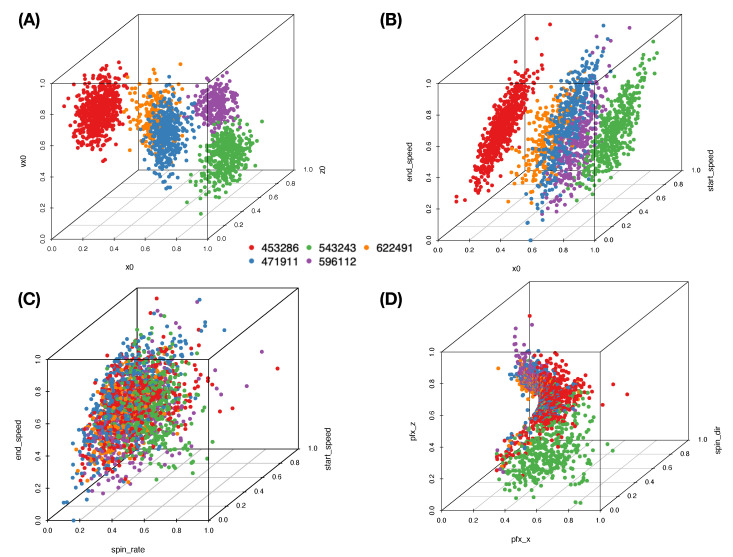
Slider’s four 3D geometries of five triplets of features (url-address for rotatable 3D plot): (**A**) {“z0”, “x0”, “vx0”}; (**B**) {“x0”, “start_speed”, “end_speed”}; (**C**) {“spin_rate”, “start_speed”, “end_speed”}; and (**D**) {“pfx_x”, “spin_dir”, “pfx_z”}. See corresponding rotatable 3D plots at https://rpubs.com/CEDA/baseball (accessed on 17 June 2021).

We first illustrate the above mechanisms within slider pitch-type. Sliders, in general, give rise to more diverse horizontal movements than curveballs and fastballs do since this pitch-type usually has a wide range of “spin_dir”, the curveball has only top-spin (top-to-bottom wheeling in the eye of the catcher), and the fastball has only back-spin (bottom-to-top wheeling). Data points of feature-triples are color-coded with respect to pitcher-IDs, as shown in Figure 5. The rotatable versions of 3D plots of the four panels can be found at https://rpubs.com/CEDA/baseball (accessed on 17 June 2021).

These four geometries pertaining to the four feature-triplets in Figure 5 (cf. https://rpubs.com/CEDA/baseball (accessed on 17 June 2021)) are evidently distinct. One natural and essential question is: How do these geometries reveal information contents of individual mechanisms? To address such a question, we first briefly point out their visible geometric structures and potential information content conveyed by the four panels of Figure 5, respectively.

**Figure 5A**  The separating point-clouds in Figure 5A indicate that the biomechanical feature-group is informative for distinguishing among pitcher-IDs. It is logical and not surprising because these features are related to pitching gestures. “x0” and “z0” are likely pitcher-specific among the set of pitchers considered here.
**Figure 5B**  The expected linear relation between “start_speed” and “end_speed” forms a shuttle shape via a cross-section view. Such shuttle compositional geometry is seemingly determined by pitcher-ID specific “x0”. Thus, this triplet of biomechanical and physical features also provides one perspective of information about pitcher-IDs. However, it is not as informative as the triplet of features in Figure 5A.
**Figure 5C**  When replacing the “x0” with “spin_rate”, the geometry becomes drastically different. We see a 3D geometry with defusing colored dots (pitcher-IDs) all over. Thus, this triplet of features is not informative for pitcher-IDs at all.
**Figure 5D**  The seemingly sharp 2D spiral manifold in $R^{3}$ implies that this triplet of features embraces one system of unknown mathematical equations that indeed depicts key physical mechanisms of pitching dynamics. Such a functional system might involve with features beyond {“pfx_x’ and “pfx_z’,“spin_dir”} since “spin_rate” is also naturally expected to play a major role in pitching dynamics. How can we explore major and minor candidate features involving in this complex system? Can we explicitly expose such involvements without mathematical formulas?

From one perspective of information content, Figure 5A,B are seemingly informative under an MCC setting because of nearly separated point-clouds with respect to (color-coded) pitcher-IDs. In contrast, Figure 5B,D are essential for the RMA setting because of both manifolds revealing evident geometric signatures. In particular, as shown via rotatable 3D plots at https://rpubs.com/CEDA/baseball (accessed on 17 June 2021), the 3D plot of feature-triplet {“spin_dir”, “pfx_x”, “pfx_z”} is appears as a sharp 2D manifold in $R^{3}$.

This 2D manifold appearance is indeed in accord with the aerodynamic phenomenon first described by German experimental scientist H. G. Magnus in 1852. This so-called Magnus effect is a force generated by a spinning object (baseball) traveling through a viscous fluid (air). The force is perpendicular to the velocity vector of the object along its trajectory. Its spin direction on the 2D plane dictates a specific combination of vertical and horizontal movements on the traveling object. That is, given the catcher’s view at the home plate, the fastball seemingly goes up against Earth’s gravity, the curveball seemingly has been pulled down harder than gravity could do, while the slider could go wild via a combination of evident vertical and horizontal movements. Other pitch-types, except the knuckleball, such as change-up, sinker, etc., are all characterized by various kinds of spin created by distinct ways of holding and releasing a baseball. The knuckleball is the only pitch-type having zero spin. Thus, it is missing spins as stabilizers. Consequently, air resistance makes the knuckleball’s trajectory very unpredictable, even for an experienced catcher.

Further, the two block-marked triplet feature-groups in Figure 4, {“spin_dir”, “break_length”,“break_angle”} and {“spin_dir”,“ax”,“az”}, also give rise to the appearance of 2D manifolds in $R^{3}$, respectively. Even though they are not as sharp as the manifold of {“spin_dir”,“pfx_x”, “pfx_z”}, these three manifolds have rather similar geometric structures. This observation is somehow surprising at first. Nonetheless, the underlying reason becomes apparent from Figure 4 since “spin_dir”, “break_angle”, “ax”, and “pfx_x” are members of the same synergistic feature-group, while “break_length”, “az”, and “pfx_z” are in another group. All these member features are commonly highly associative with “spin_dir” and “spin_rate.” These physical perspectives of understanding constitute information content and should be extracted from these manifolds via RMA. We are also intrigued by minor effects potentially exhibiting on localities of manifolds, such as pitcher-IDs, as evidently seen in Figure 5D. Such heterogeneity is indeed ubiquitous in any complex system [11]. These effects are also components of the physics of pitching dynamics. Thus, extracting information content in full from these manifolds under a RMA setting is a rather challenging issue. We attempt such a task via the first phase of CEDA developments in this paper.

## 4. Multiclass Classification (MCC)

A multiclass classification (MCC) setting can be found in the heart of a majority of real-world complex systems, such as species in nature, diseases in medicine, and pitchers in the MLB. Its collection of labels, called label space, knowingly or unknowingly, capsulizes many defining characteristics of its members through either natural or man-made labeling processes. There are two major types of labeling. One is for structured data, and the other is for unstructured data. For instance, the three Iris images in Figure 1 are unstructured data, while a 4D vector of measurements of the four features is structured data. Typical examples of unstructured data include images from nature or human activities or articles from newspapers or magazines, or even websites [12]. Labeling on unstructured data, in general, is subjective. Thus, the MCC information content of any unstructured dataset could vary from one researcher to another.

In sharp contrast, an MCC on a structured dataset is understood as a setting that the data’s curator has knowingly encoded and encapsulated the system-specific knowledge and intelligence by selecting or engineering *K* features for sufficiently labeling each of *N* subjects into one of *L* labels. The MCC information content of a structured dataset is exactly specified by the *L* labels, *K* features, and *N* subjects.

In this section, we want to computationally uncover and decode knowledge and intelligence underlying MCC settings upon a structured dataset. To a great extent, as a reverse engineering endeavor, our CEDA computational developments for MCC should uncover and decode the labeling process. That is, the ultimate goal of CEDA on an MCC is focused on its total information content. This goal has never been explicitly discussed and presented yet in the literature.

On the other hand, MCC has been a major topic in ML literature for the last two decades [13,14,15]. This ML major topic status is chiefly due to its applicability to a very wide range of real-world problems and its availability of effective predictive decision-making approaches. Recently, MCC has also become a chief apparatus for artificial intelligence (A.I.) products [16]. Thus, MCC’s ardent use is anchored primarily on its predictive capability [17,18]. In contrast with our goal in this paper, MCC’s predictive capability is just one of the essential by-products of its full information content.

### 4.1. Multiscale Complexity in MCC Information Content

In this section, we consider two structured datasets from PITCHf/x database of two pitch-types: slider and curveball. The MCC information content of each pitch-type specific structured dataset is used to shed lights on questions such as: How can we characterize chief distinctions among MLB pitchers’ pitching dynamics precisely? Can we make such characteristics and distinctions visible and explainable? Is it possible to explain each of our predictive decision-makings with a pattern-based reason? Can we make no mistakes in identifying labels? These questions are essential for understanding each of the two pitch-type specific pitching dynamic systems.

When *L*, *K*, and $N(=N_{1}+\cdots +N_{L}$), the total number of data points are not too small, and such MCC information content indeed can be rather complex. One way of perceiving such complexity is through a mixing geometry of all labels’ point-clouds determined by a subset of *k* (≤K) features. Such a mixing geometry indeed can exhibit potentially non-linear associative relations among the *k* features because their associations shape each label’s *k*-dimensional point-cloud individually and in turn dictate the global mixing geometry among all point-clouds collectively.

If one feature-set offers one perspective of mixing geometry, then there are $2^{19}-1(>$$10^{6})$ possible feature-sets. Thus, there are too many and too diverse candidate perspectives of MCC information content to be examined and chosen from. Further, due to temporal and spatial separations, these non-linear feature-to-feature associations are likely directed, so their geometric mixing patterns indeed are likely asymmetric. That is, views of one single mixing geometry can vary according to different point-clouds standpoints. As shown below, such hardly known mixing geometric asymmetry indeed helps us to see through the complexity of MCC information content, if we carefully choose which perspectives to look into.

We make use of such asymmetric mixing geometry of point-clouds to formulate the concept of complementary feature-sets. The rationale is given as follows. If a mixing geometry of one feature-set shows clear separations among a sub-group of pitchers’ point-clouds, but at the same time reveals intense mixing among another subgroup of pitchers’ point-clouds, then we would like to identify a mixing geometry of a complementary feature-set that could reveal the opposite mixing patterns. By identifying such a pair of complementary feature-sets, we can arrange them into a chain so that the uncertainty caused by mixing among point-clouds of the first feature-set can be significantly mitigated and even minimized due to different perspective views provided by the latter feature-set. Likewise, we can build a chain of three feature-groups if it is necessary. As demonstrated below, the order of feature-groups is critical in building up such a chain. A well-constructed chain of complementary feature-groups would give rise to a collection of serial patterns of mixing geometry to constitute the essential and brand new aspects of full MCC information content.

At this stage of knowledge, exploratory data analysis (EDA) could be the only computationally feasible way of discovering complementary feature-sets under an MCC setting. How to efficiently explore and discover an effective chain becomes an essential computational issue. On top of this exploring issue, another issue is how to concisely present synthesized serial pattern information from complementary feature-sets. To resolve both issues, we turn to the road-map provided by a heatmap of the K×K MCE matrix among *K* features, as illustrated in Figure 3. Due to the categorical nature of MCE, we term our data-driven explorations and computational developments the categorical exploratory data analysis (CEDA) for MCC.

Given that a feature-group specifies a mixing geometry of all point-clouds, our computational developments for CEDA for MCC pertaining to a feature-group would consist of two tasks: (1) building one label embedding tree; and (2) constructing one predictive map. Based on various feature-group specific predictive maps, the third task of our CEDA for MCC is to decide a chain of complementary feature-groups and represent the collection of serial mixing geometric patterns. The procedure of MCC is summarized in Algorithm 1.


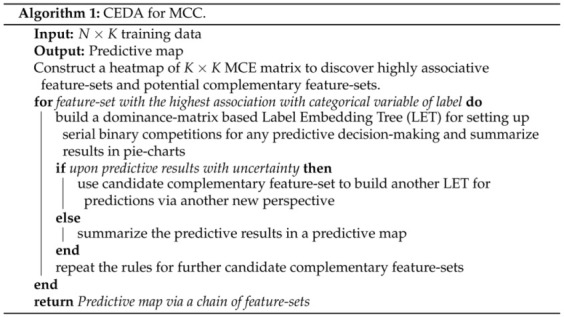


One new and significant computational development of CEDA for MCC is our proposal of “relative distance” between one label’s whole point-cloud and another label’s whole point-cloud. The geometric shapes or distributions of *L* labels’ *k*-dimensional point-clouds can be rather distinct. Such heterogeneity in geometric shape is rather difficult to be accommodated well in any direct distance measure. In other words, all direct distance measures are non-robust with respect to variations on edges of point-clouds, such as optimal transport. To robustly mitigate such edge effect, we propose a new concept of relative distance. We consider a triplet of labels’ point-clouds and intend to evaluate which two labels’ of point-clouds are closer than each of them to the third label’s point-cloud. That is, we consider “dominance”, not distance. Such closeness-based dominance is explicitly evaluated in the following collective fashion. We randomly select one *k*-dimensional point from each of the three point-clouds in $R^{k}$. We calculate the three point-point Euclidean distance in $R^{k}$, and record which the two point-point Euclidean distance dominates label-pair’s point-point Euclidean distance. We can perform these triplet competitions for as many runs as we want. For illustrative purpose, we report an experiment on the feature-group (petal-length, petal-width) of Iris data with 1000 triplet competitions.

**Figure 6:**
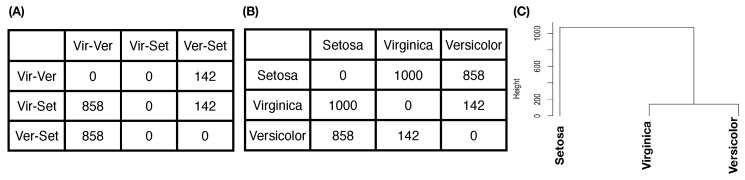
Relative distance among three Iris species’ (petal-length, petal-width) point-clouds based on 1000 triplet competitions: (**A**) label-pair dominance matrix; (**B**) relative distance matrix of three labels (species); and (**C**) label-embedding tree.

Figure 6A indicates that, among the 1000 competitions, the first row shows that the pair (Virginica, Versicolor), denoted as Vir-Ver, never dominates Vir-Set, while dominating Ver-Set 142 times. The second row shows that Vir-Set dominates Vir-Ver 858 times and Ver-Set 142 times. The third row shows that Ver-Set dominates Vir-Ver 858 times but never dominates Ver-Set. The vector row sum (142,1000,858) shows the order of closeness among the three pairs. In particular, the “relative distance” of Vir-Ver is the smallest to be dominated more than 80% of the time, while the “relative distance” of Vir-Set is the largest among the three pairwise “relative distances”. That is, we can convert the vector of row sums into a relative distance matrix of the three species of Iris, as shown in Figure 6B. It is noted that this relative distance matrix is robust with all shapes of three point-clouds [19]. Then, we can derive a HC-tree based on this relative distance matrix, as shown in Figure 6C. This HC-tree is called the label-embedding tree (LET) upon the three species.

**Figure 7:**
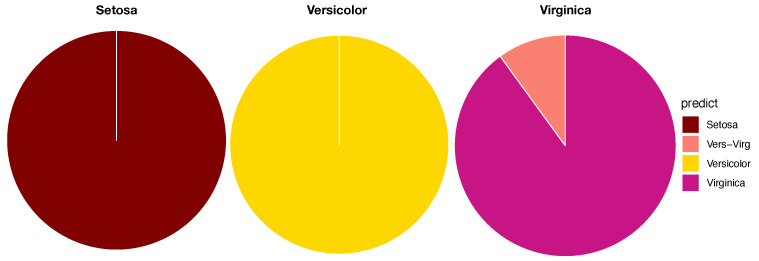
Three pie charts of the Iris example.

To ensure that the resultant LET can retain and explicitly reveal full mixing geometry among all involving point-clouds, the training dataset used for such computations usually occupies the majority of the whole dataset. In this paper, we use 80% of the original dataset as the training dataset for the first task of CEDA for MCC, while the remaining 20% is the testing dataset. In the second task of CEDA for MCC, each data point in the testing dataset is provided a series of binary competitions that are carried out by descending from the LET’s tree-top to its bottom. By continuing this Iris example with the LET given in Figure 6C, the first binary (Left-vs.-Right) competition always occurs at the top internal node of the whole LET: Left-branch (Setosa) against Right-branch (Versicolor, Virginaica). If a testing data point is declared belonging to the Left-branch, then the serial competition ends. If it is declared to be in Right-branch, then the second binary competition occurs at the top internal node of the Right-branch: Left-branch (Virginaica) against Right-branch (Versicolor). The decision-making rules of such a binary competition are given in the following subsection. There are three possible outcomes for each binary competition: (1) Left-branch; (2) Right-branch; and (3) both branches. The third outcome is designed for testing data points that are located within localities having complicated mixing patterns. Therefore, the serial competitions will end either at the bottom tree leaf or at an internal node. We use a pie chart to represent the outcomes of such serial binary competitions for testing data points having the same original label. The three resultant pie charts of the Iris example are given in Figure 7.

As illustrated via the three pie charts in Figure 7, by using (petal length, petal width) as the feature-group and its three corresponding 2D point-clouds, we can predict Setosa and Versicolor with a singleton predictor without errors. The majority of testing data points of Virginica can also afford a singleton predictor, but some testing data points must be declared with a decision as (Versicolor, Virginaica). This is an evident form of asymmetric mixing geometric pattern (see the top layer of Table 1).

To mitigate the uncertainty within such asymmetry of mixing geometry, we employ the chain of complementary feature-group. It is natural and logical to couple the feature-group (petal length, petal width) with the feature-group (sepal length, sepal width) to form a chain (petal length, petal width) ⇒ (sepal length, sepal width). The results of this chain of two complementary feature-group are shown via a two-layer table in Table 1. The effect of using (sepal length, sepal width) feature-group as a complementary feature-group (petal length, petal width) is evident. The uncertainty of Vir-Ver in the pie chart of Versicolor is explained as: this testing data point of Virginica is found close to Versicolor and Virginica, while it is found close to Setosa with respect to the perspective of (sepal length, sepal width). This is the third task of CEDA for MCC for this Iris example. This tabular representation of a collection of serial mixing geometric pattern-categories is the major part of MCC information content.

It is important to note that the ordering of feature-groups in a chain of complementary feature-groups is essential. The first feature-group plays the role of major factor in MCC, while the second and third ones are minor factors. We illustrate the importance of this ordering by computing the performance of a chain of reverse ordering (sepal length, sepal width) ⇒ (petal length, petal width). As shown in the second layers of Table 2, there are cases with uncertainty.

It is essential to recognize that such a tabular representation explicitly reveals which part of label-specific point-cloud can be perfectly predicted via a singleton-label and which parts are predicted by label-sets. Each pattern-category prescribes an error-free decision. This fact demonstrates that all inferential decisions can be fully supported by visible and explainable MCC information content. If this becomes a well-recognized fundamental standpoint for data analysis, then data analysis would be naturally embedded into scientific research.

In contrast, nowadays, the inference is the primary focus in statistics and machine learning literature [20,21,22]. Such an inferential endeavor usually focuses only on selecting the best feature-set to achieve a man-made criterion, such as minimizing the predictive error rate. Many essential parts of data’s information content pertaining to different feature-groups are ignored. Further, such an inference typically owns a foremost limit of making a singleton-label predictor. It is obvious that data do not fully support such decision-making. The consequences are seen from the following two aspects: (1) data’s pertinent uncertainty is ignored; and (2) statisticians or ML researchers force themselves to make prediction mistakes.

In the following two subsections, we expand and demonstrate our computational developments of CEDA for MCC onto two much more complex MCC settings, slider and curveball, than the illustrative Iris example.

### 4.2. CEDA for MCC on Slider Data

Here, we reiterate that the slider’s speed is slightly less than the fastball, but much higher than the curveball within a pitcher’s pitching repertoire. Its spin direction (“spin_dir”) has a much wider range than that of both fastball and curveball. Due to the Magnus effect, its horizontal movement (“pfx_x”) is very versatile. A pitcher typically changes spin directions and spin rates and speed to create an extensive range of spectrums of “pfx_x” and vertical movement (“pfx_z”) to effectively deal with batters. Hence, a professional slider pitcher is a wizard of spin and speed. The MCC on silder data is to classify among these “wizards”.

We use a slider dataset consisting of only five (=L) MLB pitchers from the 2017 season to demonstrate our CEDA for MCC computing. Here, we take a MLB pitcher-ID as a label of his slider pitches. The slider’s mutual conditional entropy matrix, as shown in Figure 4A, clearly reveals five evident blocks as marked along its diagonal. These five highly associated feature-groups with memberships are listed as follows: Group A, {“end_speed”, “start_speed”, “vyo”}; Group B, {“px”, “x”, “vz0”, “pz”}; Group C, {“z0”, “vx0”, “x0”}; Group D, {“spin_dir”, “break_angle”, “ax”, “pfx_x”}; and Group E, {“spin_rate”, “break_length”, “pfx_z”,“az”}. If we take each synergistic feature-group as a mechanical entity, we have achieve the “dimension reduction” from K=18 to 5. Via directed associations from pitcher-ID, we find the that the six highest associative features (in increasing order) are: {“x0”, “z0”, “spin_dir”, “vx0”, “ax”, “pfx_x”}.

The five computed synergistic feature-groups as five feature-sets give rise to five rather distinct mixing geometries of five pitchers’ point-clouds. Among these five feature-groups, two mixing geometries of two feature-groups are especially revealing in Figure 5A,B.

We can somehow have a glimpse of the full MCC information content under this MCC setting, mainly through their 3D rotatable plots. To precisely extract and transparently display any mixing geometry with respect to a given feature-set, our computational developments of CEDA for MCC are constructed according to the following three-step protocol.

**[MCC-Q1]**  First, based on a given feature-group and its corresponding training data subset, we derive relative closeness among all label-specific point-clouds by performing the first task of CEDA for MCC in the Iris example for all possible label triplets, and then build a feature-group label embedding tree (LET) upon the label space.
**[MCC-Q2]**  Second, we devise one universal binary competition of Left-branch-vs.-Right-branch, based $k^{*}$-nearest neighbors (KNN) [23], for all LET’s internal nodes. We perform the second task of CEDA for MCC in the Iris example for all data points in all labels’ testing data subsets, respectively. Consequently, we build a collection of *L* pie charts to represent resultant feature-group specific mixing geometries.
**[MCC-Q3]**  Third, we perform the data-driven explorations similar to the third task of CEDA for MCC in the Iris example for a chain of complementary feature-groups. Ideally, we want to discover a chain composed of the first feature-group as a major factor and followed by a short series of feature-groups as minor factors. The results of such a chain are summarized via tabular representations to reveal the collection of mixing geometric pattern-categories of various orders.

Here, as illustrated in the Iris example, a feature-group is an effective candidate for a major factor if the proportion of testing data points falling into mixing geometric categories with “certainty” is indeed as large as possible. Throughout this paper, we use 80% of observed data points for the training data subset and 20% for the testing data subset.

#### 4.2.1. Computing a Label Embedding Tree (Let)

For [MCC-Q1] in our CEDA for MCC protocol, the dimensionality of point-cloud is not an issue. However, it is a key issue for any mathematical definition of a direct distance measure between two point-clouds. For instance, researchers recently have recently applied optimal transport (OT) [24], Gromov–Hausdorff, or Gromov–Wasstein distances to evaluate a distance between two point-clouds [25,26] since such a direct measure is based on the pair’s distribution functions. Not only will the curse of dimensionality apply here, but also their non-robustness to shapes and tails behaviors of distributions will jointly worsen their effectiveness. Thus, such direct distances can be neither practical nor realistic among diverse mixing geometries. To avoid such shortcomings, it is essential to employ the concept of relative-distance or relative-closeness as stated in [MCC-Q1].

**Figure 8:**
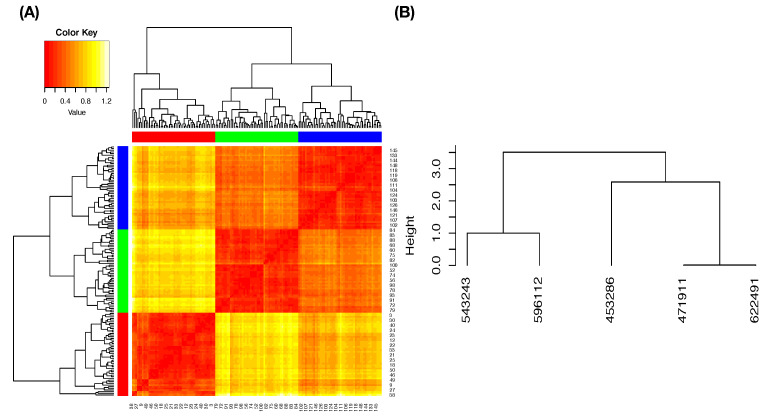
Global geometry of point-cloud based on Feature-Group C: (**A**) distance matrix of three sets of randomly selected data points from three pitchers: 453286 (red), 543243 (green) and 596112 (blue); and (**B**) LET (label embedding tree) of five pitchers.

In contrast, we demonstrate why results from [MCC-Q1] are inherently robust via the following simple experiment. We randomly select three subsets of data points from three pitchers (as color-coded) with respect to the Feature-Group C, respectively. We construct a heatmap of the distance matrix among all selected 3D data points, as shown in Figure 8A. It is seen from the heatmap that most partial ordering among all possible triplets of 3D data-points from three pitchers will likely be relatively stable; that is, the (blue, green) label-pair is most likely the smallest among the three pairs. Such inherent stochastic stability of triplet’s partial ordering would result in a robust 10×10 dominance matrix among all 10 possible pairs. Consequently, the resultant LET upon the five pitchers, as shown in Figure 8B, should be very robust.

We interpret through the tree structures of this HC tree as collectively aggregating many pieces of information of partial orderings among these five pitchers (as color-coded), such as dis(green,blue)<min(dis(green,red),dis(blue,red)) without the need of knowing the ordering between dis(green,red) and dis(blue,red). Here, dis(,) denotes the “relative-distance” defined based on a dominance matrix that reflects all possible triplet’s partial orderings, as illustrated in Figure 6A,B in the Iris example. We formally describe the algorithm for [MCC-Q1] below.

##### [Algorithm for label embedding tree (LET)]

[T-1] Given a set of *k* features, choose one triple of labels (pitchers) at a time, sample one triple of *k*-dimensional vectors from the three distinct labels’ training point-clouds, and evaluate three pairwise Euclidean distances. We only record binary partial ordering: which label-pair’s distance is dominated by the two distances of other two label-pairs.
[T-2] Repeat Step [T-1] a fixed, but large number of times across many possible triples of labels and summarize and arrange all binary partial orderings of pairwise-distance-dominance into a dominance matrix with all possible label-pairs being arranged along its row and column axes.
[T-3] Upon such a dominance matrix, which designates a row-pair dominating a column-pair, each column-sum indicates the relative-closeness of the pair of labels among all possible pairs, while each row-sum indicates the “relative-distance” of the pair of labels.
[T-4] The collection of row-sums forms a “relative-distance” matrix of all labels. A label embedding tree (LET) is built based on a HC algorithm on this dominance-based relative-distance matrix.

This algorithm is applicable for all MCC settings with structured data.

#### 4.2.2. Predictive Map of Mixing Geometric Pattern Categories

The tree geometry of LET represents a large-scale component of MCC information content. Based on LET’s binary structures from tree-top to tree-bottom, we explicitly devise a tree-descending series of “Left-branch-vs.-Right-branch” competitions at each internal nodes in this subsection. By letting each of testing data points from all labels going through such a tree-based series of competitions, we can discover the mixing geometry in very fine details. Since the serial results of competitions collectively and precisely bring out entire mixing geometry of all point-clouds (based on training data), this is an efficient way of exposing all geometric mixing patterns involving in all *L* point-clouds pertaining to *k* chosen features. Such pattern information is basically invisible when k>3. Even when k=2 or 3, such information might be hard to decipher.

For [MCC-Q2] of our CEDA for MCC protocol, we design the following algorithm to expose and extract local mixing geometric information and then summarize all results into a collection of pie charts, called predictive map. This tabular representation reveals MCC information content pertaining to a feature-group in detail.

##### [Algorithm for the Predictive Map (PM)]

**Figure 9:**
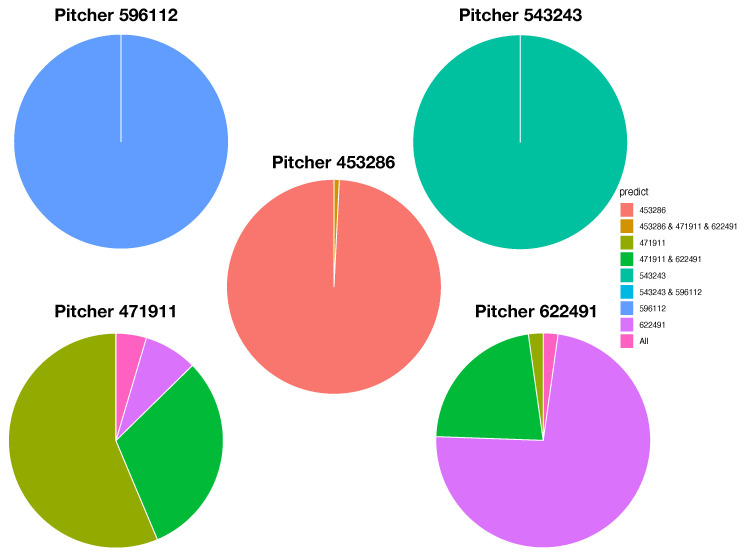
Predictive map of five slider pitchers based on Feature-Set C with threshold range [0.65, 100/65] for pseudo-likelihood values via K (=20) nearest neighbors.

**[P-1]**  Take a testing point-vector, say *x*, from the testing dataset of a label. Starting from the internal-node on the tree top of LET, we perform binary, Left-branch-vs.-Right-branch, competition via $k^{*}$—nearest neighbors of *x*. Here, $k^{*}=20$ is used. To decide which branch *x* belongs, we separate the $k^{*}$ neighbors with respect to their Left-branch and Right-branch memberships. We declare the winner according to the following policy: (a) one branch dominates in membership count; and (b) if both counts are not significantly different, then we build two membership-specific distance distributions. Upon each distribution, we calculate a pseudo-likelihood (PL) value to the median distance from *x* to all members of the same branch. We then compare the ratio of the two PL-values with respect to a threshold range $\left[C_{L},C_{U}\right]$: if the PL-ratio falls out of this range with the clear winner being Left-branch or Right-branch, then we go on to the next step; if it falls within the range, we stop the competition process and record no-winner at the internal node.
**[P-2]**  Repeat the binary competition of Step [P-1] at the winner branch’s internal node and descend further down along the winning branches until the serial of binary games stops at one no-winner internal node, which can be a branch consisting of one singleton label. Then, we record no-winner internal node’s branch members as the predicted label-set, a singleton, multiple, or none. (The case of empty predicted label-set is a device of zero-shot learning for discovering outlier.)
**[P-3]**  Repeat Steps [P-1] and [P-2] for all testing data points across all labels, and then partition each label’s testing set with respect to all its members’ predicted label-sets as categories in a pie chart, as shown in Figure 9. This feature-group specific collection of pie chart is called predictive map (PM).

On Step-[P-3], instead of using a collection of pie charts, an alternative presentation is to arranging all observed predicted label-sets across all labels along the row axis and involving labels along the column axis, all predictions of testing data points from the testing dataset are summarized into a matrix, which is also called predictive map (PM). This matrix format is employed for presenting results derived from a chain of complementary feature-groups, as shown in the subsection below.

The five pie charts, shown in Figure 9, reveal very coherent and precise mixing geometric information among the five point-clouds. The two pie charts Pitcher 596112 (purple) and pitcher-543243 (green) indicate that their point-clouds are well separated from the rest of the three point-clouds. It is nearly so for Pitcher 453286 (brown), except a small area of a mixture involving three pitchers: {453286 & 471911 &622491}. It is very significant to note that this mixture of {453286 & 471911 &622491} is uniquely pertaining to Pitcher 453286. No such mixture is found upon any other pitcher. This is a crucial piece of information content based on local scale mixing geometry. Thus, any testing data-point resulted in this pitcher-specific mixture must uniquely belong to Pitcher 453286, not the other two pitchers, Pitcher 471911 and Pitcher 622491. This is what the asymmetry of mixing geometry can offer. From a prediction point of view, we conclude that the pitches of Pitcher 453286 can be perfectly predicted with 100% precision in a singleton format. More examples of such asymmetry are reported below. This mixing geometric asymmetry-based result is at odds with all existing works in statistics and machine learning literature.

Mixing geometric information of Pitcher 471911 and Pitcher 622491 is also diverse and characteristic. More than 50% of pitches of Pitcher 471911 are standing alone and identified as Pitcher 471911, while about 75% of pitches of Pitcher 622491 are standing alone and identified as Pitcher 622491. There are about 20% pitches of Pitcher 471911 and 15% of pitches of Pitcher 622491 being identified for both pitchers, {471911 & 622491}, respectively. It is legitimate to claim that a testing data point falling into this mixture of {471911 & 622491} is predicted as (471911, 622491) because the mixing geometry fully supports such a decision. It is unnecessary or even unnatural to choose one against the other. We need to include extra information computed from different feature-sets in order to separate between these two pitchers.

In both pie charts, there are visible proportions of pitches from both pitchers: Pitcher 471911 and Pitcher 622491 are identified “wrongly”. The reason is that a pitch of Pitcher 472911 is intensively surrounded by pitches of Pitcher 622491 or vice versa. Since this mixing pattern is feature-set specific, such a mixing pattern might be altered significantly with respect to different feature-sets. This is why we need to seek for complementary feature-sets.

In sharp contrast with the above decision-making based on a predictive-map, we explicitly demonstrate why any decision-making scheme producing only singleton-predictors indeed forces us to make mistakes. Consider the choice of threshold range $\left[C_{L},C_{U}\right]=\left[1,1\right]$, which is equivalent to using a threshold value 1 on PL-ratio. By employing such a threshold, we force ourselves to choose a winning branch against a losing branch at each internal node LET. Consequentially, all predictive results under such a thresholding scheme are in the form of a singleton. It is essential to note that potential errors are to be accumulated, and, at the same time, the capability of declaring an outlier is discarded. In other words, we force ourselves to make multiple kinds of mistakes by disregarding valuable information that is indeed supported by the training data.

**Figure 10:**
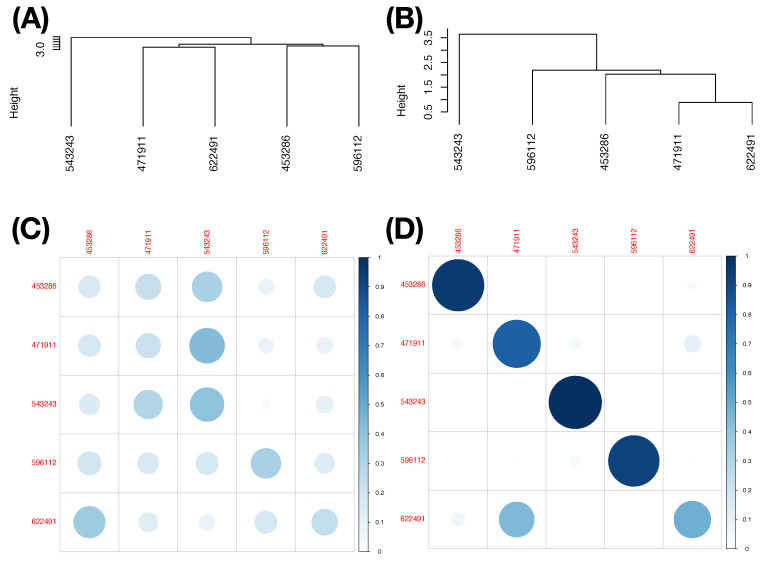
Slider’s two-label embedding tree and two predictive maps (with threshold value 1): (**A**,**C**) Feature-Set A; and (**B**,**D**) 19 features.

We illustrate results obtained under this thresholding scheme with two less informative feature-sets under Slider’s MCC setting: (1) Feature-Set A; and (2) the feature-set of all features. Their LETs and predictive maps are, respectively, reported in the two columns of Figure 10: left column (Figure 10A,C) for the Feature-Set A and right column (Figure 10B,D) for the feature-set of all features. By arranging each of the two resultant predictive maps into a matrix of proportions with row sum being equal to 1, we see all the potential mistakes across all columns. Although we still can observe the asymmetry of mixing geometries among involving point-clouds, this information is ignored completely. It is seemingly evident that the feature-group of all features is somewhat much more informative than the Feature-Set A. Thus, the potential of making mistake is less. This simple experiment is meant to demonstrate that the choice of feature-group and the asymmetry of mixing geometry are two essential components for computing MCC’s information content.

### 4.3. Slider’s MCC Information Content

After computing a label embedding tree (LET) and predictive maps (in pie chart format) for any feature-group, the third task of our CEDA for MCC is to discover an effective chain of complementary feature-sets. This chain would lead to visible and explainable MCC’s full information content. Even though we recognize the fact that the great potentials of complementary feature-sets are wide and diverse. Here, we construct a potentially effective chain by choosing a feature-group that can play the role of a major factor under this MCC setting. Since a major factor is supposed to achieve a great degree of certainty across all labels, for further building the chain, we couple this major factor with one or two minor ones.

For expositional conciseness, we also encode pitcher-IDs as follows: “a (453286)”, “b (471911)” “c (543243)”, “d (956112)”, and “e (622491)”. Our explorations leads us to the Feature-Group C as the major factor when the choice of threshold range $\left[C_{L},C_{U}\right]=\left[0.65,100/65\right]$ for PL-ratio is adopted. The five resultant pie charts for the five pitcher-labels, as reported in Figure 9, bring out the interesting fact: any distinct and unique pitcher-specific local mixing category, not sharing with any other pitchers, certainly identifies the pitcher. Further, its five pie charts are converted into a table, as shown in Table 1 (left), with respect to eight “observed” mixing geometric pattern-categories arranged and listed on its row axis. In this fashion, MCC information content from Feature-Set C is revealed and organized. The three pitchers {a, c, d} are perfectly separated to their pitcher-specific pattern-categories. The remaining four mixing geometric pattern-categories contain pitches from both Pitcher b and Pitcher e, marked by “*”. Such a pattern-category sharing means a locality of“uncertainty” among involving pitchers. For instance, the category {*b} has 49 pitches from Pitcher b and 1 pitch from Pitcher e, while the category {*e} has 7 pitches from Pitcher b and 33 pitches from Pitcher e.

For [MCC-Q3], we further explore fine-scale MCC information content by exploring potential feature-sets for roles of minor factors. This exploration is performed by dissecting the above localities having uncertainty (under the major factor) as looking through a different perspective via a feature-group. We choose the feature-group {“x0”, “z0”, “spin_dir”} for this minor role. Its pie chart is summarized and presented in Table 3 (right) with seven categories that are subject to uncertainty. In Table 4, the four asterisk-marked mixing geometric pattern-categories via Feature-Set C are projected with respect to six mixing geometric pattern-categories (without {a}). From its first three columns for {*b}, we see that the 49 pitches of Pitcher b are exclusively divided into three second-order categories: 34 in {*b-b}, 14 in {*b-be}, and 1 in {all(abcde)}. One pitch from Pitcher e is alone in {*b-e}. Therefore, these 50 pitches are exclusively located. This is a sense of complementary feature-sets.

In contrast, the 40 pitches in category {*e} are divided into two second-order categories: 25 in {*e-e} (with 1 pitch belonging to Pitcher b and 24 belonging to Pitcher e) and 15 in {*e-be} (6 from Pitcher b and 9 from Pitcher e). Therefore, these two second-order categories are subject to uncertainty and need further explorations from another perspective of another feature-group. Likewise, second-order categories are derived from first-order categories {*be} and {*all}. We choose the Feature-Set A–C, including members of three feature-groups A, B, and C as the third feature-group in the chain. Pattern-categories of mixing geometry of Feature-Set A–C are given in Table 5.

All the third-order mixing geometric pattern-categories are listed in Table 6. This table shows promising results. There are 15 out of 21 triplet categories obtaining certainty regarding being exclusively in Pitcher b or Pitcher e. This phenomenon manifests clear characters of a chain of third-ordered complementary feature-groups.

As for pitches belonging to the other five categories of third order, geometrically speaking, intensive mixing between Pitcher b and Pitcher e is found within these localities. Therefore, it is reasonable and necessary to make a prediction decision as {Pitcher b, Pitcher e} within these localities. On the other hand, making any singleton-based decision upon such localities is firmly against the evidence supported by the data. Surely, odds-ratios should accompany the predictive label-sets.

In summary, with a carefully selected chain of three complementary feature-groups, the slider dataset’s MCC information content is explored in-depth and collectively represented by its LET and a series of ordered predictive maps. There are at least three notable merits of such MCC information content. The chief one is that the first, second, and third orders of mixing geometric pattern-categories jointly provide the basis for understanding the labeling’s rationales. The second merit is that they offer a platform for error-free decision-making. That is, all predictive results are fully supported by patterns embraced by the data. The third merit is that such serial tabular mixing geometric pattern-categories allow us to dissect the uncertainty of unexplainable black-boxed results derived from popular machine learning algorithms, such as random forest and various boosting approaches. This merit is somehow surprising, as demonstrated after the following subsection, in which we demonstrate the same characters of MCC information content of the curveball dataset.

### 4.4. Curveball’s MCC Information Content

We report here our CEDA for MCC computational results on the curveball dataset. We employ exactly the same computational algorithms and exploring themes to work out curveball’s MCC information content. The selected chain of complementary feature-groups turns out to be even more efficient in this MCC setting than that of the slider.

The curveball is a pitch-type with top-spin, which is the opposite of a fastball’s back-spin. Unlike fastball, generating top-spin is a bit unnatural. Thus, a curveball pitch in general is much slower than a fastball or slider for any MLB pitcher. Its typical trajectory has a significant vertical drop (measured by “pfx_z”) when reaching the home plate. This drastic drop is caused by the Magnus effect being added onto the gravity force. It is a somewhat effective pitch-type in dealing with batters. In sharp contrast with the slider, its horizontal movement (measured by “pfx_x”) has a narrow range centering around zero.

The curveball dataset used here consists of seven (=K) MLB pitchers. We compute its 19×19 mutual conditional entropy matrix, as shown in Figure 4B. Its heatmap reveals six evident blocks as marked along its diagonal. These six highly associated feature-groups are: Group A, {“px”, “x”}; Group B, {“vz0”, “pz”}; Group C, {“spin_dir”, “break_angle”, “ax”, “pfx_x”}; Group D, {“break_length”, “pfx_z”,“az”}. Group E, {“end_speed”, “start_speed”, “vyo”}; and Group F, {“ay”, “spin_rate”, “z0”, “vx0”, “x0”}. We also calculate the directed associations of the 19 features toward the label space and rank them. The six highest associations (in increasing order) are: {“x0”, “z0”, “spin_dir”, “start_speed”, “vy0”, “break_angle”}. This feature-set of six is denoted as BB.

**Figure 11:**
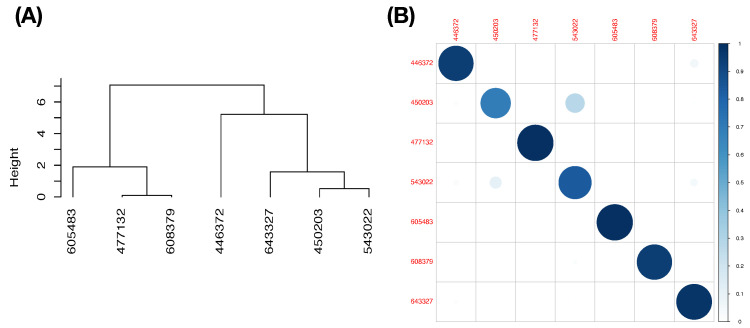
Curveball’s MCC setting with threshold range [1,1] with respect to feature-group: DEF: (**A**) label embedding tree of feature set DEF; and (**B**) predictive graphs and predictive matrix.

With the same 4-to-1 ratio for training-to-testing data subsets, our CEDA follows the same CEDA for MCC protocol as we previously have done under slider’s MCC setting. Our choice of a chain of complementary feature-groups begins with a feature-group, denoted as DEF, including members from three feature-groups: D–F (Figure 4B). As the major factor of the chain, the LET of feature-set DEF and its predictive map with the thresholding scheme with [1,1] are given in Figure 11A,B, respectively. Once again, such a predictive map shows asymmetry of mixing geometries and the amounts of errors by forcefully committing to a singleton label-predictor.

In contrast, with respect to feature-group DEF and the choice of threshold range $\left[C_{L},C_{U}\right]=\left[0.65,100/65\right]$ for PL-ratio, the resultant predictive map is reported in Table 7. This feature-set DEF seems rather efficient. We see that, except Pitcher b (with ID: 450203), the other six pitchers are nearly exclusively predicted. Pitcher-IDs are encoded as following: Pitcher a (446372), Pitcher c (477132), Pitcher d (543022), Pitcher e (605483), Pitcher f (608379), and Pitcher g (643327). Among the 12 mixing geometric pattern-categories of the first order, six are pitcher-ID specific, while six ategories have uncertainty. Next, we choose the feature-group BB to be the second feature-group as a minor factor in the chain to further explore the fine-scale MCC information content.

With the chain of two feature-groups, DEF to BB, such chain results are reported in Table 8. The majority of the second-order mixing geometric pattern-categories turn out to be pitcher specific, while those with uncertainty have relatively extreme odds-ratios. We indeed can explore further. We stop here to avoid repeating similar messages regarding the effectiveness of such chains of complementary feature-groups. The above two tables of local mixing geometric pattern-categories demonstrate the effects of two complementary feature-sets, and at the same time jointly reveal the curveball’s MCC information content. Such a chain of two feature-groups offers perfect predictive decision-making with explainable supporting evidence. Likewise, the platform consisting of the first and second orders of mixing geometric pattern-categories can map out machine learning methodologies’ uncertainly, as discussed below.

### 4.5. Dissecting Uncertainty of Results from Machine Learning Algorithm

Under MCC settings, ML algorithms, such as random forest [27] and various boosting approaches [28,29], are popularly employed. They can achieve low classification error rates. Such successes are mainly seen when the number of features *K* is large relative to the size of label space *L*. Many factors certainly have contributed significantly to such successes. However, all these decisions are derived from black-boxes in the sense of no reasons and interpretations attached. This issue of non-interpretability is a serious one from the perspectives of real-world applications as well as sciences, since, even if they can potentially achieve very low error rates, these error rates are not equal to zeros. That is, all these machine learning results under any MCC setting are subject to the uncertainty of being simultaneously right and wrong.

Indeed, our MCC information content can serve as an ultimate standard of validity for uncertainty resulting from machine learning algorithms. The validity check is subject to each testing data point to both CEDA and any machine learning algorithm. Then, we view and check which one of the mixing geometric pattern-categories a testing data point’s ML decision falls into. Such a check leads to one of the following four possibilities, with a ML decision landing in the mixing geometric pattern-category:

**1.** **[certainty–coherent]:** with certainty, and they are coherent;
**2.** **[certainty–incoherent]:** with certainty, but they are incoherent;
**3.** **[uncertainty–coherent]:** with uncertainty and they are coherent; and
**4.** **[uncertainty–incoherent]:** with uncertainty, but they are incoherent.

The certainty–coherent case serves as a confirmation of the ML-decision. Both cases of certainty–incoherent and uncertainty–incoherent likely point out that the ML’ predictive result is“definitely” wrong. There exists still uncertainty when a ML predictive result falls into uncertainty–coherent case. However, it will be 100% correct if we report our predictive decision based on the mixing geometric pattern-category. Upon these four cases, it seems that all ML methodologies can evidently be significantly strengthened by projecting their predictive results onto our MCC information content. At the same time, we resolve the interpretation issue completely.

We illustrate such conclusions through applying random forest on the slider and curveball datasets. Our experiments applied random forest multiple times and two versions of boosting multiple times. All results are relatively consistent with the above conclusions. Thus, we only report results from the random forest.

In Table 9, we see that random forest (RF) makes 13 errors: 2 from Pitcher b being assigned to Pitcher e, 1 from Pitcher c being assigned to Pitcher b, and 10 from Pitcher e being assigned to Pitcher b. For the first-order mixing geometric pattern-categories, the certainty–incoherent error of Pitcher b prediction on one pitch of Pitcher c has been confirmed at the Category c. Among 12 remaining errors going into the second-order mixing geometric pattern-categories in Table 4, we see that one RF’s error is confirmed in the Category b–e in Table 10. The 11 remaining errors going into the third order mixing geometric pattern-categories in Table 6, we see two pitches of Pitcher e are confirmed in e–e–be and e–e–abe; the other nine pitches are in [uncertainty–coherent] case: two from Pitcher b and seven from Pitcher e, are all in mixing geometric pattern-categories with uncertainty.

Such a dissection on the uncertainty of Random Forest’s errors brings out the fact that all its errors are likely coming from rather intensive mixing localities of the geometry of all involving point-clouds. From this perspective, it would be advantageous to make ML predictions with projections into mixing geometric pattern-categories of MCC information content. This conclusion is proper throughout all experiments via two versions of boosting as well.

To iterate such a conclusive statement, we again view the errors made by random forest on the curveball dataset from the perspective of its MCC information content. There are 14 errors committed by random forest on one application, as shown in Table 11. In Table 12, all 14 errors fall into first-order mixing geometric pattern-categories with uncertainty, as shown in Table 7. Further, in Table 12 and due to one (d–bd) and one (bdg–db) being corrected, 12 out of 14 fall into the second-order mixing geometric pattern-categories, as shown in Table 8. Likewise, most errors from the two versions of boosting approaches are also found in an MCC’s mixing geometric pattern-category with uncertainty. Again, we reiterate that it would be advantageous to make ML predictions coupled with projections into mixing geometric pattern-categories of MCC information content.

## 5. Response Manifold Analytics (RMA)

Some features and feature-groups evidently do not shed informative lights on labels’ idiosyncratic characteristics, such as {“ax”, “az”, “pfx_x”, “pfx_z”, “break_length”, “break_angle”}. Together with “end_speed”, these features are measured near the home plate. They are response variables in nature. In contrast, the rest of the 12 features, including those informative features for labels’ characteristics, are all measured around the pitcher’s mound. They are covariate variables in nature. Thus, the separation of response-vs.-covariate variables is physical along both spatial and temporal axes. The spatial axis is 60${}^{\prime }$-6${}^{\prime \prime }$ (18.39 m) in length from the pitcher’s mound to the home plate, while the temporal axis is about 0.5 s (500 ms) in time-span for a baseball to arrive home plate (at 90 mph).

The collective associative relations of 7-response-to-12-covariate are contemplated as pitching’s aerodynamic mechanics. The data analysis study of this physical mechanics as one whole system is termed response manifold analytics (RMA). That is, RMA must embrace and discover large- and fine-scale associative relations that are universally governed by Newton’s laws of forces, including gravity, speed, pressure, and Magnus effects of spin in aerodynamics. Many biomechanical factors are also involved because they are responsible for force and spin. Due to their underlying functional structures, such multiscale associations are contained in and revealed through manifolds in Euclidean spaces.

Even though it is somehow unthinkable to attempt the task of synthesizing these individually known forces into one complex system of pitching dynamics involving all tremendous or even overwhelming details, and further no known existing functional systems could embrace all such intertwined and coupled forces with all fine-scale details, all data analysts understand that the PITCHf/x database likely captures such a functional system largely, if not entirely. Moreover, they also believe that it is just not enough to study such pitching dynamics only according to Newton’s principles. Thus, our challenge in the second part of this paper is: Can we approximate such a complex functional system purely based on data analysis?

To approximate a complex functional system, we again adopt the CEDA theme: by using of all features’ categorical nature, we perform global-to-local and coarse-to-fine multiscale explorations onto response-to-covariate associative relations. In procedural terms, our CEDA for RMA computations first identifies a group of features as a major factor that accommodates the known physical principles of the complex system under study and then discovers all potential features of minor factors that reveal heterogeneity within response-to-covariate associations. In terms of rationale, our CEDA for RMA developments makes use of the following geometric basis. The chosen group of covariate features of major factors would couple with a targeted group of response features to form an evident geometric manifold with low complexity. Upon such a manifold, the collection of rectangles or hypercubes jointly framed by major covariate features’ categorical structures is taken as a collection of localities on the manifold. These localities contain relatively understandable and straightforward associative structures regarding response features toward major covariate features and heterogeneous and detailed associative structures regarding response features toward minor covariate features. By discovering all these major and minor associative structures, we indeed link such locality-specific structures into a manifold-based approximation of the entire functional system. This is our CEDA for the RMA theme for computing RMA information content. The algorithm for RMA is summarized in Algorithm 2.
 **Algorithm 2:** CEDA for RMA.  **Input**: Training data  **Output**: Predictive inferences  Search for one major factor (a covariate feature-set) for the response feature-set  based on the MCE roadmap and build a geometry of response manifold by coupling response and major factor feature-sets.  Partition the entire response manifold into a collective of hypercubes as localities.  Identify a minor factor based on Shannon entropy within each locality.  Find localities containing the vector of a major factor pertaining to testing data  and predict each locality by incorporating information provided by major factor  and extra information from minor factor.

**Figure 12:**
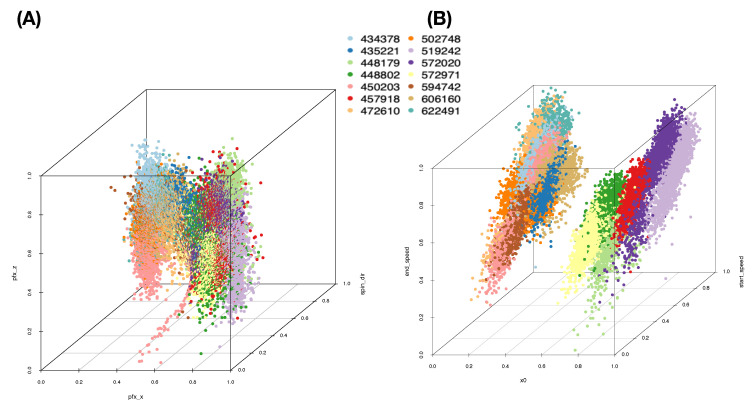
Fastball’s two manifolds: (**A**) {“pfx_x”, “pfx_z”, “ spin_dir”}; and (**B**) {“x0”, “start_speed”, “end_speed”} (see corresponding rotatable 3D plots at https://rpubs.com/CEDA/baseball (accessed on 17 June 2021)).

In this section, we develop our CEDA for RMA computations on the fastball data theme with a focus placed on two intrinsically distinct manifolds, as shown in Figure 12, which are primary mechanisms of pitching dynamics of the fastball.

### 5.1. Manifolds on {“pfx_x”, “pfx_z”, “ spin_dir”}

From aerodynamics, we know that the Magnus effect is indeed underlying the seeming 2D manifolds of {“pfx_x”, “pfx_z”, “ spin_dir”} in $R^{3}$, as vividly demonstrated through rotatable 3D plots, slider and fastball (https://rpubs.com/CEDA/baseball (accessed on 17 June 2021)), with their snapshots shown in Figure 5D and Figure 12A, respectively. The two manifolds show obvious differences in geometric characteristics of pitch-types. Mechanically speaking, it means that Magnus effects operate in distinct manners within distinct domains of feature values with respect to the two pitch-types. Mathematically, the two systems of functions describing these 2D manifolds are highly nonlinear across the entire span of pitching dynamics. Hence, it would be reasonable to explore a pitch-type specific functional system.

**Figure 13:**
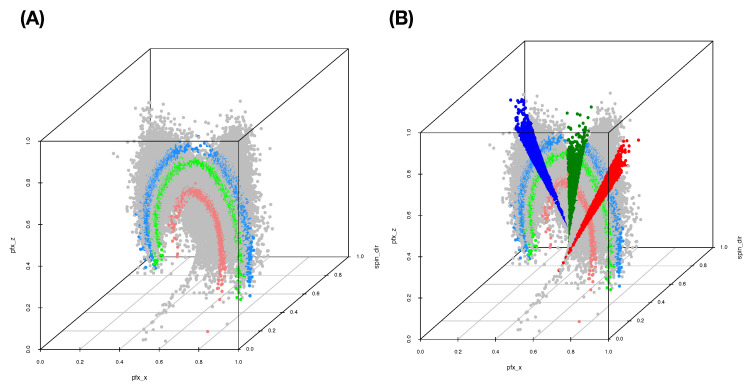
Manifolds of {“pfx_x”, “pfx_z”, “spin_dir”} with marked strips: (**A**) three strips of “spin_rate”; and (**B**) three strips of “spin_rate” intersecting with three strips “spin_dir” (see corresponding rotatable 3D plots at https://rpubs.com/CEDA/baseball (accessed on 17 June 2021)).

**Figure 14:**
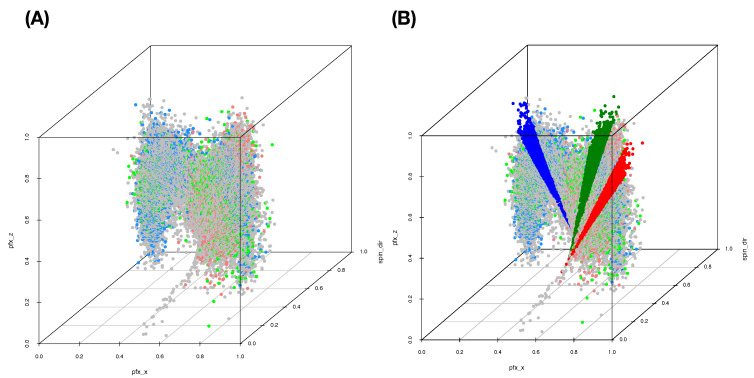
Manifolds of {“pfx_x”, “pfx_z”, “spin_dir”} with marked strips: (**A**) three strips of “start_speed”; and (**B**) three strips of “start_speed” intersecting with three strips “spin_dir” (see corresponding rotatable 3D plots at (https://rpubs.com/CEDA/baseball (accessed on 17 June 2021)).

For expositional simplicity, our computational developments of CEDA for RMA in this section are illustrated through the fastball. We begin fastball’s RMA by explicitly exploring which major covariate features jointly determine response features {“pfx_x”, “pfx_z”} among 12 covariate features plus the categorical one of pitch-IDs. Upon the discovery of 2D manifolds of {“pfx_x”, “pfx_z”, “ spin_dir”} in $R^{3}$, as shown in Figure 12A, we know that “ spin_dir” is one major covariate feature. We seek other major covariate features by color-coding each candidate feature’s histogram with respect to bins and then marking them onto the manifold of {“pfx_x”, “pfx_z”, “ spin_dir”}. The idea is that each bin of major covariate feature will mark a clear-cut strip onto this manifold. For example, the bins of “ spin_rate” mark clear-cut strips, as shown in Figure 13A. However, it is not the case for “ start_speed”, as shown in of Figure 14A. The are no clear-cut strips but defused colored points all over the manifold. This defusing pattern indicates that“ start_speed” is not a major feature of this manifold.

If we also mark three strips of “ spin_dir” from three separate bins of its histogram, we see nine more or less rectangular intersecting patches, as shown in Figure 13B. Therefore, all bins of the histogram of “ spin_dir” coupled with all bins of the histogram of “ spin_rate” frame a lattice of patches. Each patch in this patching lattice is a locality of {“ spin_dir”,“ spin_rate”,“pfx_x”, “pfx_z”} 4D manifold. After discovering a set of major features and constructing a patching ensemble, we aim to approximate the entire manifold as one whole. This approximation scheme is seen as one of the chief merits of using a major covariate feature to understand how targeted response features come to be as they are. It also becomes clear that the defusing patterns induced by including a non-major feature, such as “ start_speed”, as seen in Figure 14, cannot afford reasonable approximations across the entire range of targeted response features.

**Figure 15:**
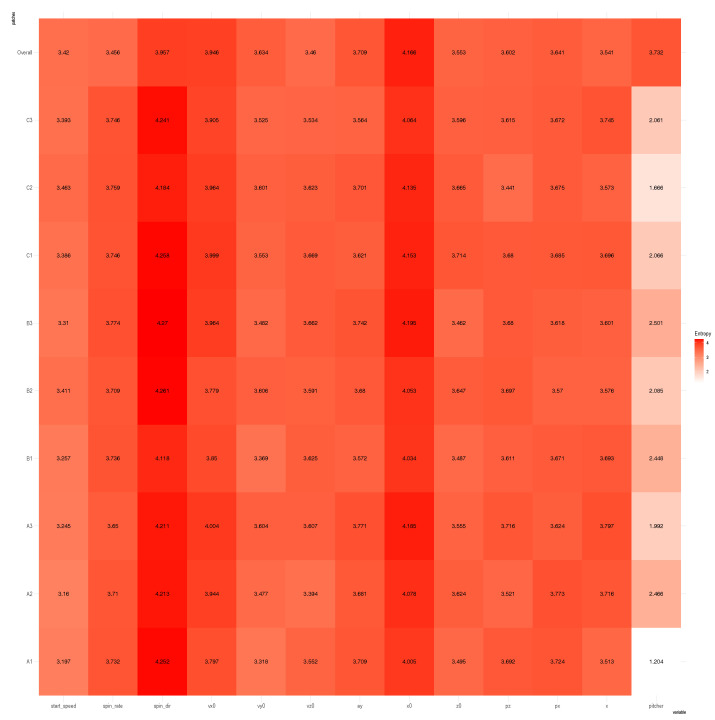
Minor features selection based Shannon entropy on nine patches on manifolds of {“pfx_x”, “pfx_z”, “spin_dir”} framed by nine intersecting patches via three strips of “spin_dir” with three strips “spin_dir”, as seen in Figure 13.

After discovering the {“spin_dir”, “spin_rate”} being a set of major features to the target response features {“pfx_x”, “pfx_z”}, the resultant lattice of localities of 4D manifold becomes the basis for exploring all potential minor features. It is worth reiterating that a minor feature could reveal its effects on some localities while having no effects at all on the rest of localities. One illustrative example feature is the pitcher-ID. As revealed in Figure 12A, some pitcher-IDs dominate some localities, and some are intensively mixed on some other localities. Therefore, it is essential to figure out and document which features’ bins are dominant on which localities. Shannon entropy can naturally bring out degrees of such kind of dominance of bin-memberships. We report the Shannon entropies for the nine patches identified in Figure 13B across all covariate features and Pitcher-ID in Figure 15. We see that the pitch-ID is the obvious minor feature. Such a finding is categorical.

The effort of identifying all potential minor features within each locality of 4D manifold {“spin_dir”, “spin_rate”, “pfx_x”, “pfx_z”} is crucial for seeing the spectrum of RMA information content contained in PITCHf/x about pitching dynamics of fastball. Collectively, all minor features’ heterogeneous effects across all localities become a map of heterogeneity pertaining to all relevant minor features. This map helps major features to provide very detailed information about MLB pitching dynamics from the perspective of response features {“pfx_x”, “pfx_z”}.

In summary, on the task of figuring out how Magnus effects works out fastball’s {“pfx_x”, “pfx_z”}, the discovered major covariate features {“spin_dir”, “spin_rate”} give us a global view as well as local view through the 4D manifold. The identified minor features reveal their roles within the localities of this manifold. This major-to-minor is meant for global-to-fine scales of information content. This theme echoes Nobel laureate physicist P. W. Anderson’s statements in his 1972 paper with the title “More is different”: the twin difficulties of scale and complexity (heterogeneity) are keys to understanding large complex systems.

We also discover two more manifolds: {“spin_dir”, “ax”, “az”} and {“break_length”, “break_angle”,“spin_dir”}. It is not surprising that both manifold are very similar with the manifold {“pfx_x”, “pfx_z”, “ spin_dir”} because, as shown in the mutual conditional entropy matrix in Figure 4, {“spin_dir”, “ax”, “pfx_x”, “break_angle”} form a tightly associative feature-group, as do {“spin_rate”, “az”, “pfx_z”, “break_length”}. Therefore, we do not need to repeat the same RMA for response features {“ax”, “az”, “break_length”, “break_angle”}.

The above computational results of CEDA for RMA imply that our predictive decision-making for {“pfx_x”, “pfx_z”}, {“ax”, “az”} or {“break_length”, “break_angle”} must be performed following this inferential protocol: (1) identify a proper locality framed by the major covariate features; (2) choose a proper and focal neighborhood system within this locality, such as $k^{*}$-nearest neighbors; and (3) use each identified minor covariate feature as a sieve to filter out incoherent neighbors. We are confident that this intrinsic global-to-local idea underlying this CEDA for RMA-based prediction protocol is universal for all complex systems. We discuss the performance of such an inferential protocol in the next subsection.

### 5.2. Predictive Inferences via a Manifold on {“pfx _X”, “Pfx _Z”, “ Spin _Dir”}

After identifying major and minor feature to multiple response features, it is natural to discuss the the computational task of predictive inference. This discussion further demonstrates the merits of our CEDA for RMA through revealing the manifold’s impacts on predictive decision-making.

Let a target set of response variables be {“pfx_x”, “pfx_z”}, denoted as $\left\{Y_{1},Y_{2}\right\}$. Upon 4D manifold of {“pfx_x”, “pfx_z”, “spin_dir”, “spin_rate”}, we identify a set of major covariate features: {“ spin_dir”, “ spin_rate”}, denoted as $\left\{X_{1},X_{2}\right\}$, and a minor feature “pitcher-ID”, denoted as $Z_{1}$. How to predict $\left\{Y_{1}^{*},Y_{2}^{*}\right\}$ given $\left\{x_{1},x_{2},z_{1}\right\}$. Since features “pfx_x” and “pfx_z” are evidently associated with each other. A reasonable predictive decision-making mechanism estimates such a bivariate response variable $\left(Y_{1}^{*},Y_{2}^{*}\right)$ simultaneously. According to the above inferential protocol, our predictive decision-making process is given as follows.

Identify a two-dimensional training-covariate-rectangle $B<x_{1},x_{2}>=B\left[X_{1}\right]\times B\left[X_{2}\right]$, where $x_{1}\in B\left[X_{1}\right]$ and $x_{2}\in B\left[X_{2}\right]$ with $B[X_{1}]$ and $B[X_{2}]$ being one bin of the two histograms of features $X_{k}$ with k=1,2 based on training data, respectively. In addition, we denote the training-response-region of $B<x_{1},x_{2}>$:

$$F\left[B<x_{1},x_{2}>\right]=\left\{\left(Y_{1},Y_{2}\right)|\left(X_{1},X_{2}\right)\in B<x_{1},x_{2}>\right\}.$$

That is, $F\left[B<x_{1},x_{2}>\right]\times B<x_{1},x_{2}>$ is a patch (so-called locality) shown in Figure 13 on the manifold {“pfx_x”, “pfx_z”, “ spin_dir”,“ spin_rate”}. This locality is the proper region of interest that captures the global scale of pattern information upon the manifold on an identified local covariate region of $B<x_{1},x_{2}>$. Our predictive decision-making on $\left(Y_{1}^{*},Y_{2}^{*}\right)$ is proceeded by applying the $k^{*}$-nearest neighbor approach with $k^{*}=20$. Let the set of $k^{*}$ nearest neighbors be denoted by $B<x_{1},x_{2}>=\left\{{\left(X_{1},X_{2}\right)}^{h}\in B|h=1,2,...,20\right\}$. Next, we define $B^{z_{1}}<x_{1},x_{2}>$ as a subset containing members of $B<x_{1},x_{2}>$ that are actually associating with the observed minor feature $z_{1}$.

Then, we define a focal subset of $F[B<x_{1},x_{2}>]$ given $\left(x_{1},x_{2},z_{1}\right)$ as:

$$F\left[B^{z_{1}}<x_{1},x_{2}>\right]=\left\{\left(Y_{1},Y_{2}\right)|\left(X_{1},X_{2}\right)\in B^{z_{1}}<x_{1},x_{2}>\right\}.$$

Then, we make a predicted vector $\left({\hat{Y}}_{1}^{*},{\hat{Y}}_{2}^{*}\right)$ as the center of mass, or simple average, of focal subset $F[B^{z_{1}}<x_{1},x_{2}>]$.

In this manner, this estimation of $\left({\hat{Y}}_{1}^{*},{\hat{Y}}_{2}^{*}\right)$ indeed incorporate global and local structures of major and minor covariate features. We see this predictive decision-making simultaneously embraces geometric patterns of manifold {“pfx_x”, “pfx_z”, “ spin_dir”,“ spin_rate”} that capture the multiscale associations between bivariate response s{“pfx_x”, “pfx_z”} and bivariate major covariate {“ spin_dir”, “ spin_rate”} and takes care of heterogeneity due to minor feature “pitcher-ID” within locality.

Here, we experiment by comparing our CEDA for RMA-based predictive decision-making with Random Forest regression. Here, Random Forest regression is applied to estimate $Y_{1}^{*}$ and $Y_{2}^{*}$, separately to all covariate 12 features. The values of tuning parameters used in Random Forest regression are all default choices. We consider three kinds of sum of errors: (1) individual sum of squared errors for $Y_{1}^{*}$(“pfx_x”) and $Y_{2}^{*}$(“pfx_z”) (see Table 13); (2) bivariate correlated sum of squared error with respect to overall training covariance matrix of $\left\{Y_{1},Y_{2}\right\}$, see Table 14; and (3) bivariate correlated sum of squared error with respect to $B<x_{1},x_{2}>$ locality-specific training covariance matrix of $\left\{Y_{1},Y_{2}\right\}$ (see Table 15). Here, the bivariate correlated sum of squared errors is calculated by re-scaling the bivariate error vector with respect to its corresponding covariance matrix, which is either based on all training data of $\left\{Y_{1},Y_{2}\right\}$, or locality-specific $\left\{Y_{1},Y_{2}\right\}$ corresponding to identified rectangles $B<x_{1},x_{2}>$.

Across these three tables, our CEDA for RMA-based predictive decision-making overall significantly outperform Random Forest Regression on eight of nine patches marked in Figure 13 on the manifold {“pfx_x”, “pfx_z”, “ spin_dir”} in terms of individual response features as well as a bivariate vector. Both approaches are equal in the C1 patch. The bivariate correlated sum of squared error to the overall training covariance matrix is particularly outstanding, upon which we see merits on our inferential protocol. It is noted that patch-wise covariance matrix re-scaling might be too volatile, as seen in Table 15.

### 5.3. Manifold on {“x0”, “Start _Speed”, “End _Speed”}

The response feature “end_speed” seemingly involves the simplest mechanism among the seven response features. Its functional relationship with “start_speed” or “vx0” (the releasing speed) is naturally expected to be linear. Thus, feature “start_speed” is an obvious major feature toward “end_speed”. Are there other major features? It is not “spin_dir” or “spin_rate”. Indeed, we have not found any other than the feature “x0”. Different ranges of “x0” values seemingly lead to different pitcher-specific scatter plots of “start_speed” against “end_speed”. It is indeed more-or-less so for all these pitch-types, as shown in Figure 5C and Figure 12B, respectively, for slider and fastball. Further, it is noted that the feature “x0” is highly associative with pitcher-ID, as discussed in the MCC section. It is natural to see that pitcher-ID is a minor feature, given “x0” is taken as a major feature.

Upon the manifold of {“x0”, “start_speed”, “end_speed”}, we explore pitcher-specific relations of {“x0”, “start_speed”} to response feature “end_speed” by considering the simple linear regression model fitting to each individual pitcher’s data is:

$$Y_{i}=\alpha +\beta_{1}X_{1,i}+\beta_{2}X_{2,i}+\varepsilon_{i}$$

with $Y_{i}$, $X_{1,i}$, and $X_{2,i}$ for *i*th pitch’s “end_speed”, “x0”, and “start_speed”, respectively.

All pitcher-specific estimates of $\beta_{2}$, the slope value of “start_speed”, are shown in Table 16. These statistically significant estimates collectively have a range [0.783,1.099] of values, which is much wider than slider and curveball’s narrow ranges centering around 1.0. This is striking because it is somehow against our intuition. The reasons underlying these pitchers’ exceptional low values of $\beta_{2}$ needs further explanations and explorations. On the other hand, this manifold on {“x0”, “start_speed”, “end_speed”} gives rise clear message of inferential importance of major features as well as the minor ones. This involvement of pitcher-ID make the RMA connected to MCC. We need first to identify candidate pitcher-IDs for prediction purposes and then apply the linear regression inference. This is exactly in accord with our CEDA for RMA-based inferential protocol.

## 6. Conclusions from MCC and RMA Perspectives

Under the setting of quantitative covariate features, we develop computational algorithms and protocols of CEDA for MCC and RMA to extract data’s multiscale information contents with heterogeneity. Such resultant information contents echo physicist P. W. Anderson’s universal view on large complex systems. By fully reflecting the geometries and manifolds underlying complex systems of interest, we demonstrate that the chief merits of our computational developments are geared for system understanding. Upon three relatively small datasets taken from the PITCHf/x database, we also point out that inferential decision-making based on data’s information content is likely universally valid. Hopefully, this conclusive message can bear essential impacts on any other system studied far beyond MLB’s pitching dynamic systems.

On the front of CEDA for MCC, our developments of a chain of complementary feature-groups and tabular representations of the resultant collection of mixing geometric pattern-categories explicitly reveal asymmetry of serial mixing geometries as MCC’s information content. Such discoveries and synthesis reiterate the essence of CEDA for MCC in discovering diverse perspectives of heterogeneity within any dataset from a complex system. Our partial ordering and dominance matrix concepts for computing a label embedding tree (LET) are robust and efficient. Consequently, With LET’s reliable binary geometry on a label space, our serial binary competitions with length of order $O(\log_{2}K)$ are an effective way of exploring complex mixing geometry among many high dimensional point-clouds. It is worth mentioning that it is to the great benefit of ML’s MCC results by coupling with results of CEDA for MCC because its certainty and uncertainty are documented and explained.

On the front of CEDA for RMA, we show that manifolds based on response-to-covariate associations in Euclidean spaces are efficient foundations for studying complex systems. Such manifolds pave the platform for figuring out data’s information content with locality-based heterogeneity and facilitate fundamentally efficient and straightforward inferential protocols, which are particularly suited for predictive decision-making on multiple response variables.

At the end of this section, we remark that the second phase of CEDA development is undergoing research in a separate study. This phase of CEDA would have the capacity of accommodating categorical covariate features and other complex settings. After the second phase of development, CEDA can become one of the foundations of data analysis on a structured data matrix. As such, CEDA would be a standard protocol for studying complex physical systems. That is, data analysis might realistically become a scientific discipline on its own, as John Tukey projected in 1962.

## Figures and Tables

**Table 1:** Petal-pair ⇒ Sepal-pair.

	Setosa	Versicolor	Virginica
setosa	10	0	0
versicolor	0	10	0
virginica	0	0	9
vv-setosa	0	0	1

**Table 2:** Sepal-pair ⇒ Petal-pair.

	Setosa	Versicolor	Virginica
setosa	9	0	0
versicolor	0	6	0
virginica	0	0	7
setosa-virginica	0	0	1
virginica-setosa	1	0	0
virginica-versicolor	0	2	0
All-versicolor	0	1	0
vv-versiocolor	0	1	0
vv-virginica	0	0	2

**Table 3:** Predictive maps of two feature-sets: C (left); and {“x0”,“spin_dir”,“z0”} (right).

Feature-Set-C	a	b	c	d	e	{x0, spin_dir z0}	a	b	c	d	e
a	123	0	0	0	0	a	124	0	0	0	0
*b	0	49	0	0	1	b	0	43	0	0	2
c	0	0	91	0	0	c	0	0	85	1	0
d	0	0	0	56	0	d	0	0	0	52	0
*e	0	7	0	0	33	e	0	3	0	0	26
a, b, e	1	0	0	0	0	b, e	0	40	0	0	17
*b, e	0	27	0	0	10	a, b, c, d, e	0	1	6	3	0
*all	0	4	0	0	1						

**Table 4:** Predictive results of chain: feature-sect-C to feature-set {“x0”,“spin_dir”,“z0”}.

*b	b	e	*e	b	e	*be	b	e	*all	b	e
b	34	0	b	0	0	b	6	2	b	3	0
c	0	0	c	0	0	c	0	0	c	0	0
d	0	0	d	0	0	d	0	0	d	0	0
e	0	1	e	1	24	e	2	1	e	0	0
be	14	0	be	6	9	be	19	7	be	1	1
all	1	0	all	0	0	all	0	0	all	0	0

**Table 5:** Predictive map of feature-set: A–C.

ABC-	a	b	c	d	e
a	122	0	0	0	0
b	0	34	0	0	1
c	0	0	89	0	0
d	0	2	1	56	0
e	0	0	0	0	15
abe	2	0	0	0	4
be	0	44	0	0	19
all(abcde)	0	7	1	0	6

**Table 6:** Predictive results of chain: feature-sect-C to feature-set {“x0”,“spin_dir”,“z0”} to feature-set A&B&C.

Chain of 3 Sets	b	e	Chain of 3 Sets (cont’d)	b	e
e-e-b	1	1	be-be-d	1	0
e-be-b	1	0	be-b-be	2	1
e-e-abe	0	4	be-be-be	12	6
e-e-be	0	6	be-b-all	0	1
e-be-be	5	5	be-e-all	1	1
e-e-all	0	1	be-be-all	2	1
e-be-all	0	1	all-b-b	1	0
be-b-b	4	0	all-be-be	1	0
be-e-b	1	0	all-b-all	2	0
be-be-b	4	0	all-be-all	0	1

**Table 7:** The predictive map matrix from feature-set DEF. (*) marks categories with uncertainty.

DEF	a	b	c	d	e	f	g
a	146	0	0	0	0	0	1
*b	0	10	0	2	0	0	0
c	0	0	80	0	0	0	0
*d	0	4	0	30	0	1	0
e	0	0	0	0	44	0	0
f	0	0	0	0	0	54	0
*g	1	0	0	0	0	0	135
cef	0	0	2	0	0	0	0
*bd	0	111	0	10	0	0	0
*bdg	0	1	0	5	0	0	4
*abdg	13	3	0	1	0	0	4
all	0	0	0	0	0	4	0

**Table 8:** Classification results of chain of two feature-sets: DEF to BB.

*b	a	b	d	g	*d	a	b	d	g	*g	a	b	d	g
b	0	5	1	0	d	0	2	20	0	a	1	0	0	0
abdf	0	1	0	0	f	0	0	2	0	f	0	0	0	1
bd	0	4	1	0	abdf	0	2	0	0	g	0	0	0	91
					bd	0	0	2	0	bdf	0	0	0	2
					bdf	0	0	4	0	all	0	0	0	41
					all	0	0	2	0					
*bdg	**a**	**b**	**d**	**g**	*abdg	**a**	**b**	**d**	**g**	*bd	**a**	**b**	**d**	**g**
b	0	1	0	0	a	11	0	0	0	b	0	12	1	0
d	0	0	3	0	g	0	0	0	3	d	0	2	4	0
g	0	0	0	1	abdf	2	2	1	0	abdf	0	8	0	0
bd	0	0	2	0	bd	0	1	0	0	bd	0	89	5	0
bdf	0	0	0	1	bdf	0	0	0	1					
all	0	0	0	2										

**Table 9:** Results of one random forest application on slider data with errors indicated.

RF	a	b	c	d	e
a	124	0	0	0	0
b	0	85	1	0	10
c	0	0	90	0	0
d	0	0	0	56	0
e	0	2	0	0	35

**Table 10:** Locations of random forest’s errors on slider with respect to the: first-order predictive map (left); second-order predictive map (middle); and third-order predictive map (right).

RF	b	c	e	RF	b	e	RF	b	e
b	0	0	1	b-e	0	1	e-be-be	2	0
c	0	1	0	e-e	0	2	be-b-be	0	1
e	2	0	2	e-be	2	0	be-b-all	0	1
be	0	0	6	be-b	0	2	be-be-be	0	3
all	0	0	1	be-be	0	4	be-be-all	0	1
				all-be	0	1	all-be-all	0	1

**Table 11:** Results of one random forest application on curveball data with errors indicated.

RF	a	b	c	d	e	f	g
a	158	0	0	0	0	0	1
b	2	128	0	9	0	0	0
c	0	0	82	0	0	0	0
d	0	1	0	37	0	0	0
e	0	0	0	0	44	0	0
f	0	0	0	0	0	59	0
g	0	0	0	2	0	0	144

**Table 12:** Locations of random forest’s errors on curveball with respect to the: first-order predictive map (left); and second-order predictive map (right).

RF	a	b	d	RF	a	b	d
b	0	0	2	b-b	0	0	1
d	0	0	1	b-bd	0	0	1
bd	0	1	6	bd-b	0	0	1
bdg	0	0	1	bd-d	0	0	1
abdg	2	0	1	bd-bd	0	1	4
				bdg-bd	0	0	1
				abdg-abdf	2	0	1

**Table 13:** Sum of squared error (SSE).

	Ours		RF	
**Patch**	**pfx_x**	**pfx_z**	**pfx_x**	**pfx_z**
A1	0.00062	0.00061	0.00072	0.00110
A2	0.00093	0.00266	0.00088	0.00149
A3	0.00016	0.00192	0.00080	0.00246
B1	0.00098	0.00271	0.01796	0.00410
B2	0.00188	0.00560	0.00138	0.00476
B3	0.00182	0.00375	0.00087	0.00546
C1	0.00103	0.00306	0.00212	0.00197
C2	0.00090	0.00296	0.00053	0.00677
C3	0.00136	0.00283	0.00050	0.00241

**Table 14:** Bivariate correlated sum of squared errors (covariance matrix Σ computed based on all training nodes).

Patch	Ours	RF
A1	0.024	0.599
A2	0.061	0.071
A3	0.012	0.467
B1	0.034	0.639
B2	0.940	1.230
B3	0.078	5.884
C1	0.053	0.005
C2	0.079	8.244
C3	0.024	0.307

**Table 15:** Bivariate correlated sum of squared errors (nine locality-specific covariance matrixes Σ computed based on only training within each locality).

Patch	Ours	RF
A1	14.852	123.574
A2	6.657	11.377
A3	2.366	35.105
B1	13.855	77.308
B2	70.931	58.974
B3	15.402	305.538
C1	7.416	0.456
C2	11.795	332.379
C3	3.333	23.411

**Table 16:** Fastball.

Pitcher	Intercept	x0	Start_Speed	Residual std Error	df
572971	0.038 *	−0.046 *	1.009 *	0.027	1491
457918	0.124 *	−0.028	0.785 *	0.037	1759
606160	0.012	−0.136 *	0.981 *	0.042	1242
519242	0.025	−0.039	0.928 *	0.038	1649
450203	0.067 *	−0.279 *	0.934 *	0.033	1627
572020	0.247 *	−0.220 *	0.870 *	0.036	1713
502748	0.085 *	−0.011	0.801 *	0.032	1028
448179	0.021	−0.037	0.916 *	0.036	1288
435221	0.146 *	−0.045	0.783 *	0.031	692
448802	0.072 *	−0.011	0.861 *	0.037	1470
434378	0.018	−0.112 *	0.931 *	0.044	1988
472610	−0.024 *	0.382 *	0.952 *	0.035	698
594742	0.065 *	−0.005	0.825 *	0.031	746
622491	−0.006	−0.172 *	0.982 *	0.039	922

Note: * means significant at α = 0.05.

## Data Availability

The pitching data are available in the PITCHf/x database belonging to Major League Baseball via http://gd2.mlb.com/components/game/mlb/ (accessed on 17 June 2021).
